# ANN-based analysis of MHD third-grade hybrid nanofluid flow over a thin needle with fuzzy volume fraction under nonlinear radiation and heat generation

**DOI:** 10.1038/s41598-025-29013-2

**Published:** 2025-12-03

**Authors:** Imran Siddique, Muhammad Nadeem, Bushra Shakoor, Habib Kraiem, Abdullatif Saleh Ghallab, Zaher Mundher Yaseen, Barno Abdullaeva

**Affiliations:** 1 Department of Mathematics, University of Sargodha, Sargodha, 40100 Pakistan; 2 Mathematics in Applied Sciences and Engineering Research Group, Scientific Research Center, Al-Ayen University, Nasiriyah, 64001 Iraq; 3 Federal University of Technology Parana UTFPR, Mechanical Engineering Department DAMEC, Postgraduate Program in Mechanical and Materials Engineering PPGEM, Research Center for Rheology and Non-Newtonian Fluids CERNN, Curitiba, PR 81280-340 Brazil; 4 Center for Scientific Research and Entrepreneurship, Northern Border University, 73213 Arar, Saudi Arabia; 5 Department of Computer Science, University of Science and Technology, 13064, Sana’a, Yemen; 6 Civil and Environmental Engineering Department, King Fahd University of Petroleum and Minerals, 31261 Dhahran, Saudi Arabia; 7 Department of Mathematics and Information Technologies, Tashkent State Pedagogical University, Tashkent, Uzbekistan

**Keywords:** Third-grade fluid, Thin needle, HNF, MHD, TFN, ANN, Engineering, Mathematics and computing, Nanoscience and technology

## Abstract

This investigation examines the magnetohydrodynamic (MHD) flow and heat transfer characteristics of a third-grade hybrid nanofluid (HNF) containing ${\mathrm{Al}}_{{2}} {\mathrm{O}}_{{3}}$ and ${\mathrm{TiO}}_{2}$ nanoparticles suspended in sodium alginate over a horizontally translating thin needle under the influence of viscous dissipation, nonlinear thermal radiation, and internal heat generation effects. The governing partial differential equations (PDEs) are transformed into ordinary differential equations (ODEs) via similarity transformations and solved numerically using the bvp4c finite-difference method. For uncertainty analysis and comparison through the triangular membership function, the volume fractions of nanosized materials have been taken as triangular fuzzy numbers (TFNs) [0, 10%, 20%]. The TFNs are controlled with the help of the $\zeta {\text{ - cut}}$ approach. Additionally, Artificial Neural Networks (ANNs), trained via Bayesian Regularization (BRS) and Levenberg–Marquardt (LMS) algorithms, are developed for predictive modelling of skin friction and heat flux coefficients. Parametric analysis reveals that the flow gradient decreases as the volume fraction and the magnetic factor increase. The thermal gradient of the liquid and HNF rises with larger values of the thermal ratio factor, volumetric fraction, and Eckert number. The HNF demonstrates superior thermal performance compared to conventional third-grade fluid. According to the fuzzy analysis, the HNF exhibits the highest thermal energy transmission compared to both nanofluids (NFs). The ANN models show excellent agreement with numerical solutions, exhibiting low mean squared errors (MSE) across training, testing, and validation datasets. This integrated fuzzy-neural framework provides a novel approach for uncertainty quantification in HNF applications, with direct relevance to biomedical devices, aerospace thermal management, and renewable energy systems.

## Introduction

The usage of non-Newtonian liquids is becoming a more common cause of growing applicability in various industrial sectors such as oil-water emulsions, coal in water, china clay, paint production, suspension fabrication, cosmetic creams, sewage sludge, pharmacology, food processing, and polymer synthesis. The nature of non-Newtonian liquids has been demonstrated using a variety of liquid models, including integral type, rate type, and differential type. The liquid of third-grade (Differential type liquid) may predict both normal stresses and shear thickening/thinning spectacles. Many scientists have studied the motion of third-grade liquid from various perspectives^1–3^. The flow of an unsteady third-grade liquid was investigated by Keceba and Yürüsoy^4^, and the solution was found using similarity variables. Abbasbandy and Hayat^5^ calculated an analytical solution for a specific liquid of third grade. The MHD differential type liquid was examined by Hayat et al.^6^ across a stretched cylinder. Hatami et al.^7^ investigated the effect of gold nanosized materials on a third-grade flowing liquid through a porous tube.

Many researchers have evaluated thermal and flow analysis in a moving thin needle with various flow problems due to the significance of these applications in industries and transportation, such as hot wire anemometers, acetone craft, microscale cooling systems, blood flow problems, thermal energy transmission apparatuses, and boats. Using an incompressible viscous liquid, Lee^8^ explored boundary layer flow around a horizontally oriented needle. This study also examines the numerical results and asymptotic characteristics. Chen and Smith^9^ conducted an analytical investigation into forced convective thermal energy transmission in non-uniform, incompressible fluid flow over a non-isothermal thin needle, providing fundamental insights into the thermal boundary layer behavior under variable thermal conditions. The flow on a thin needle, which is comparable to a stirring flow, was shown using the finite-difference technique by Ishak et al.^10^. Nonlinear thermal radiation’s effect and double diffusion on Casson NFs flow along thin needles were established by Souayeh et al.^11^. Almeida et al.^12^ present a mathematical study of unsteady mixed convection flow of a Casson-Williamson nanofluid over a curved, melting, stretching sheet, incorporating thermal radiation, Joule heating, chemical reactions, and second-order slip at the surface. Subsequently, Nayak et al.^13^ examined the influence of Ohmic heating, viscous dissipation, and variable buoyancy forces on nanofluid flow over a thin needle, offering valuable insights into the interplay of thermal and electromagnetic Impacts in microscale flow systems. With thermal radiation and inside nanosized-material transport, Chu et al.^14^ researched the effect of homogeneous–heterogeneous reactions on the thin needle. Mabood et al.^15^ employed the shooting method to analyze the thermal behaviour and Arrhenius activation energy (AAE) in the flow of a micropolar fluid over a horizontally moving thin needle. Upreti et al.^16^ analyze how nanoparticle shape influences boundary‐layer flow and heat transfer of Au–TiO₂/ethylene glycol hybrid nanofluid over a thin needle, including the effects of magnetic field, temperature‐dependent viscosity, thermal conductivity, and quadratic convection/radiation. Additional noteworthy contributions addressing various physical phenomena associated with thin needle configurations are documented in studies^17–24^.

Traditional liquids (oil, glycol, water, ethylene, etc.) are frequently used in industrial and practical applications. These liquids, however, have a poor heat transmission rate due to their low thermal conductivity. The basic liquids are supplemented with a single type of nanosized material known as a nanoliquid. The nanosized materials enhance the thermal efficiency of the working liquid, such as domestic refrigerators, power generators, electronic devices, manufacturing paper, nuclear reactors, the automotive industry, and air conditioning. Choi and Eastman^25^ initially familiarized the notion of NFs in 1995, claiming that when metallic nanosized (100 nm) particles are mixed into host liquids, then thermal conductivity is significantly enhanced. Hayat et al.^26^ calculated the stagnation point and variable heat flux for NFs over the thin horizontal needle. The Tiwari–Das model was used, Salleh et al.^27^ inspected heat transmission, and the mixed convection flow over the thin needle. Afridi et al.^28^ examined the combined Impacts of chemical reactions, internal heat generation, and NF flow over a slender needle, providing important insights into reactive transport phenomena in microscale geometries. Shah et al.^29^ inspected the behavior of CNTs (SWCNTs & MWCNTs) and bioconvection phenomena on Maxwell NFs with entropy rate. Theoretically directed the Williamson NFs flow over a moving thin needle by Khan et al.^30^. Bilal et al.^31^ predicted the outcome of thermal conductivity, fluctuating thermal radiation, and MHD Williamson NFs across a stretching cylinder. In efforts, a large amount of work on NFs under thin needles has been examined^32–36^.

An HNF is a material that contains two different nanometer-sized particles in the base liquid. For better thermal efficiency and rate of heat transmission, HNFs have been used in many applications such as heat control, localized refrigerators, electronic devices, welding, hydroelectric processing, vehicle thermal control, paper production, nuclear sectors, and microelectronics. Sulochana et al.^37^ examined the menthol-based HNF using a stirring needle. Devi & Devi^38,39^ investigated the thermal energy transmission and flow of HNFs $\left( {{\mathrm{Al}}_{{2}} {\mathrm{O}}_{{3}} {\text{ + Cu/H}}_{{2}} {\mathrm{O}}} \right)$ through a stretched sheet. Subhani and Nadeem^40^ calculated the behaviour of HNF $\left( {{\text{Cu + TiO}}_{{2}} {\mathrm{/H}}_{{2}} {\mathrm{O}}} \right)$ through a stretching sheet. Over a stretched cylinder, Yousefi et al.^41^ considered the stagnation point flow that uses water with Copper-Titania as an HNF. Takabi and Salehi^42^ considered the calculation of natural convection, heat source, and thermal energy transmissions in the ${\mathrm{Al}}_{{2}} {\mathrm{O}}_{{3}} {\mathrm{/H}}_{{2}} {\mathrm{O}}$ nanoliquid and $\left( {{\mathrm{Al}}_{{2}} {\mathrm{O}}_{{3}} {\text{ + Cu/H}}_{{2}} {\mathrm{O}}} \right)$ HNF. In the literature, there is a piece of additional information that is well presented in^43–47^.

The magnetic appearances of an electrically conducting liquid, such as seawater, plasmas, liquid metals, and electrolytes, are studied in magnetohydrodynamics (MHD). It is also found in a variety of equipment like generators, power pumps, heat exchangers, and electrostatic filters. Magnetic gradients generate the Lorentz forces in a flowing liquid. It reduces the flow of liquid while increasing the thermal. As a result, magnetic gradient inclusion is critical in postponing boundary layer separation. Khan et al.^48^ analyzed the upshot of MHD, hall current, and viscous dissipation on a thin stirring needle. Saqib et al.^49^ explored the MHD stream of an HNF in terms of nanosized-material structure, heat production, and thermal radiation. Ramzan et al.^50^ scrutinized the impression of the Christov-Cattaneo heat flux model, MHD, and heterogeneous-homogeneous reactions on third-grade liquid flow. MHD hybrid $\left( {{\mathrm{Al}}_{{2}} {\mathrm{O}}_{{3}} {\text{ + Cu/H}}_{{2}} {\mathrm{O}}} \right)$ NFs flowing to the porous stretched/shrunken sheet at non-linear velocity were observed by Zainal et al.^51^. For example^12,52–54^, a large number of investigations on MHD and thermal energy transmission have been done.

The relevant factors and variables are meant to be crisp or defined accurately in the mainstream study of physical systems represented using differential equations. However, these characteristics may be regarded as ambiguous and uncertain in reality due to the possibility of mistakes in observations and experiments. Due to limitations in measurement precision and inherent variability, many physical systems lack complete or precise information about governing factors and boundary conditions. This uncertainty can compromise the reliability of deterministic models, necessitating the incorporation of fuzzy logic, which gives rise to fuzzy differential equations (FDEs) for a more realistic system representation. Here, the FDEs are applied to compare the fuzzy volume fraction of the nanomaterial based on a triangular membership function. Chang and Zadeh^55^, and the concept of FDEs by Kandel and Byatt^56^ introduced FD. The concepts of fuzzy derivative and integration were introduced by Seikkala^57^. Uniqueness and existence theorem for a solution of FDE was studied by Kaleva^58^. Abbasbandy and Allahviranloo^59^ proposed a numerical solution for solving the FDEs. Jameel et al.^60^ examined the comparison between finite difference and Newton’s methods using second-order fuzzified boundary value problems. Borah et al.^61^ employed fractional derivatives, specifically the Caputo–Fabrizio and Atangana–Baleanu operators, to investigate the behavior of MHD second-grade fluids under a fuzzy environment. By utilizing the Zadeh extension principle along with TFNs, the non-dimensional governing equations were transformed into fuzzified counterparts to account for uncertainty in physical factors. Building on this framework, Barhoi et al.^62^ explored the Impacts of second-order velocity slip and MHD flow of a viscous fluid over a porous shrinking surface within a fuzzified context, further highlighting the relevance of fuzzy modeling in complex fluid dynamics. FDM was used by Hazarika et al.^63^ to study the viscous liquid for free convection and MHD flow over an infinite vertical channel in a fuzzy environment. Nadeem et al.^64^ recently explored ohmic heating and MHD flow on third-grade liquid along an inclined channel in an uncertain environment. Furthermore, various researchers have employed FST to accomplish well-known technical and scientific conclusions^65–70^.

Artificial Neural Networks (ANNs) have come to occupy a prominent place in solving intricate nonlinear problems of fluid dynamics. In contrast to traditional numerical methods, ANNs possess a wider potential for convergence capabilities, allowing them to model complex fluid behaviors with greater flexibility. Their intrinsic potential to learn independently without having to be programmatically reconfigured for each set of problems provides for successful modeling of highly nonlinear systems and data reconstruction in cases of missing or lost data with considerable accuracy. Additionally, ANNs have high accuracy and adaptability, making them particularly well-suited for problems where conventional analytical or numerical methods are inapplicable. In the past decade, artificial intelligence (AI) in the form of ANNs has emerged as an integral tool in various scientific and engineering disciplines, including fluid mechanics. The incorporation of AI within this gradient is essential in order to improve our comprehension and predictive potential with respect to fluid flow patterns, turbulence characteristics, and other dynamic fluid processes. These improvements find real-world application in industries such as aerospace and automotive engineering, climate modelling, and environmental studies. The growing popularity of ANN-based models and probabilistic approaches in computational engineering also follows from their strength, computational simplicity, and capacity to provide robust solutions in complicated fluid systems. Raja et al.^71^used neural network-based intelligent computing to describe entropy generation in MHD third-grade NF flows under chemical reactions and viscous dissipation, suitably bringing out the nonlinear intricacies of such systems. Upreti et. Al^72^. employed ANN-LMS to predict skin friction coefficient and local Nusselt number with high accuracy by examining TiO₂–Al₂O₃/kerosene hybrid nanofluid flow over a permeable non-flat plate under magnetic field, mixed convection, Joule heating, and nonlinear thermal radiation. Kumar et al.^73^ deployed LMS-ANN to investigate how nanolayer morphology influences magnetized micropolar nanofluid flow over the microchannel using the ANN feed-forward backpropagation technique. Almeida et al.^74^ investigated the flow characteristics of a magnetized liquid over an elongating curved sheet using a neural network model trained via the LMS optimization algorithm. Kumar et al.^75^ developed a machine learning framework employing the same LMS-based algorithm to analyze Prandtl fluid flow over a curved sheet. Kumar et al.^76^ developed an ANN to model time-dependent radiative Casson fluid flow with couple stresses in a microchannel, focusing on transient velocity, temperature fields and entropy analysis.

The integration of artificial intelligence, particularly ANN with fuzzy logic, presents a powerful paradigm for modelling complex and uncertain systems. Recent studies have begun to explore these methodologies in fluid flow contexts. Divya et al.^77^ examine the unsteady flow of a Sisko tri-hybrid nanofluid over a rotating disk in a porous medium using Adaptive Neuro-Fuzzy Inference System and Reptile Search Algorithm ANFIS-RSA modelling. Their model achieves over 98 % accuracy in predicting skin friction, velocity components, and Nusselt number, suggesting that nanoparticles may enhance control in cancer therapy via thermal effects. Sundar and Mewada^78^ employed the multilayer perceptron (MLP) and adaptive neuro-fuzzy inference system (ANFIS) models to investigate the exergy efficiency, frictional entropy, and thermal entropy production in $\mathrm{CoFe}2\mathrm{O}4$/Water nanofluids. Kulkarni and Chinchanikar^79^ developed both ANN and ANFIS models to predict cutting force, surface roughness, and tool life in turning Inconel 718 under minimum quantity lubrication with a hybrid nanofluid (50-50 Al₂O₃ and MWCNT in palm oil). Their results show ANFIS outperforms ANN in predicting surface roughness (4.5% error vs 11%) and tool life (6% vs 10%), though ANN better predicts cutting force.

The primary purpose of this work is to see how MHD affects hybrid nanosized materials and third-grade liquid flowing via a horizontally rotating slender needle. Also, the influences of viscous dissipation, heat source, and nonlinear radiation are involved. For simulating the flow problem using the Tiwari–Das model^47^, Sodium Alginate is considered as the host liquid, while alumina (${\mathrm{Al}}_{{2}} {\mathrm{O}}_{{3}}$) and Titanium dioxide (${\mathrm{TiO}}_{2}$) as the hybrid nanosized materials. Using the notable similarity variables, the pair of PDEs is modified into ODEs. The built-in MATLAB bvp4c technique is executed for the numerical solution. We achieved outstanding findings that are consistent with past research. All of these studies are dependent on a crisp volume fraction value [0.01–0.04.01.04], which may be perceived as uncertain to simulate the real situation. In this way, the volume fraction of (${\mathrm{Al}}_{{2}} {\mathrm{O}}_{{3}}$ and ${\mathrm{TiO}}_{2}$) materials is in the arrangement of fuzzy numbers. The triangular membership function is constructed using fuzzy numbers through the application of the $\zeta {\text{ - cut}}$ technique, which facilitates the representation of uncertainty in parameter estimation by defining lower, modal, and upper bounds. The novel integration of fuzzy triangular numbers with artificial neural network (ANN)-based schemes introduces a distinctive framework for managing uncertainty in data analysis, particularly within the context of complex fluid dynamics. This methodological innovation enhances the robustness and interpretability of computational models, allowing for a better understanding of intricate fluid phenomena. Here, we discussed the comparison of NFs and HNFs through a triangular membership function. The reasons for doing this evaluation led to the following research questions:

(i) What does the thermal efficiency improve when viscous dissipation, thermal radiation, and heat source/sink impacts are present?
(ii) Which nanosized materials provide the most effective cooling for the thin needle?
(iii) How does volume percentage impact flow and thermodynamic properties in a current flow system?
(iv) Whose NFs combination is responsible for the amplified heat flux?
(v) What effect do fuzzy volume percentages have on flow and heat characteristics in a present system?
(vi) Can ANN frameworks serve as efficient surrogates for real-time prediction of thermal and hydrodynamic behavior in advanced engineering systems using HNF?
(vii) What advantages does the integration of artificial intelligence and fuzzy logic offer for modeling complex nonlinear fluid dynamics over classical approaches?

## Formulation of the problem

**Fig. 1:**
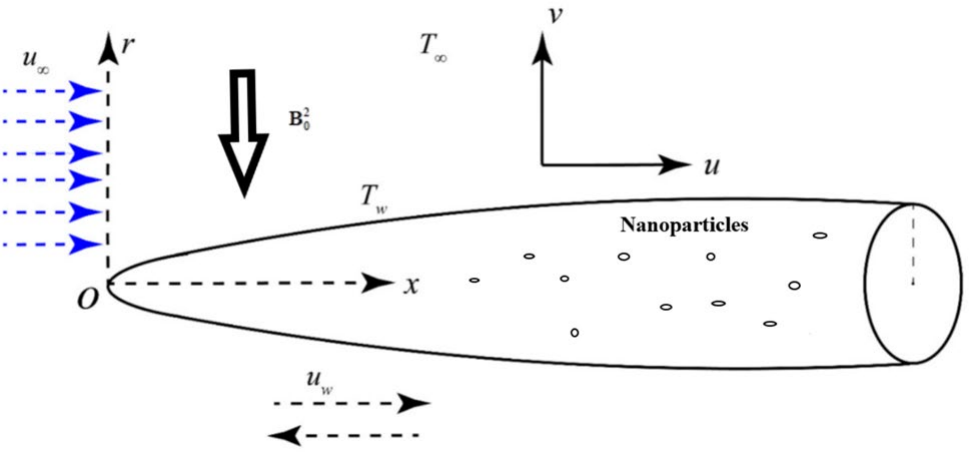
Physical flow geometry.

This study considers a laminar, steady, axisymmetric flow of an incompressible third-grade HNF containing $\mathrm{Al}_2\mathrm{O}_3$ and $\mathrm{TiO}_2$ nanoparticles suspended in sodium alginate around a horizontally oriented thin needle in a cylindrical coordinate system $(x, r, \varphi ),$ where the needle surface is defined by $r = R(x) = \sqrt {\frac{{\chi xv_{f} }}{U}} ,$ is immersed in a viscous fluid with kinematic viscosity $v_{f}$ and the composite flow velocity is represented by $U = u_{w} + u_{\infty } ,$ as shown in Fig. 1. The thin needle approximation is rigorously justified through the slenderness parameter $\varepsilon = {{R(x)} \mathord{\left/ {\vphantom {{R(x)} x}} \right. \kern-0pt} x} < < 1$, ensuring the needle radius remains small compared to the axial distance, while the boundary layer thickness $\delta (x) \approx \sqrt {\frac{{xv_{f} }}{U}}>> R(x)$ validates the boundary layer approximation with Reynolds number based on needle radius ${\mathrm{Re}} = {{UR} \mathord{\left/ {\vphantom {{UR} {\nu_{f} < < 1}}} \right. \kern-0pt} {\nu_{f} < < 1}}$ guaranteeing viscous force dominance over inertial effects. The mathematical framework employs axial symmetry ${\partial \mathord{\left/ {\vphantom {\partial {\partial \varphi = 0}}} \right. \kern-0pt} {\partial \varphi = 0}}$, steady-state conditions ${\partial \mathord{\left/ {\vphantom {\partial {\partial t = 0}}} \right. \kern-0pt} {\partial t = 0}}$, and incompressible flow assumptions $\left( {\nabla .\,{\mathbf{V}} = 0} \right)$ validated by Mach number $Ma < < 1$, with the radial momentum equation reducing to ${{\partial p} \mathord{\left/ {\vphantom {{\partial p} {\partial r \approx 0}}} \right. \kern-0pt} {\partial r \approx 0}}$ due to the scale analysis, where axial velocity $u \approx U$, radial velocity $v \approx \varepsilon U < < u$, and curvature effects become negligible when ${R \mathord{\left/ {\vphantom {R {\delta < < 1}}} \right. \kern-0pt} {\delta < < 1}}$.

A uniform magnetic gradient of strength $B_{0}$ is applied in the radial direction, introducing MHD Impacts, while the axial pressure gradient is neglected due to the slender geometry of the needle. The needle surface is maintained at an elevated thermal, inducing a thermal boundary layer in the surrounding liquid. The energy balance incorporates the influences of internal heat generation or absorption (modelled as a heat sink/source term), nonlinear thermal radiation, and viscous dissipation. These combined Impacts play a crucial role in determining the thermal and hydrodynamic behaviour of the non-Newtonian hybrid nanofluid flow near the needle.

Based on the aforementioned assumptions, the governing boundary layer equations, including the continuity, momentum, and energy equations, are formulated in cylindrical coordinate systems to accurately describe the axisymmetric behavior of the flow and thermal gradients around the thin needle^4–6,10,24,27^.

1 $$\frac{\partial }{\partial r}\left( {rv} \right) + \frac{\partial }{\partial x}\left( {ru} \right) = 0,$$

2 $$\rho_{hnf} \left( {v\frac{\partial u}{{\partial r}} + u\frac{\partial u}{{\partial x}}} \right) = \mu_{hnf} \left( {\frac{{\partial^{2} u}}{{\partial r^{2} }} + \frac{1}{r}\frac{\partial u}{{\partial r}}} \right) + \beta_{3} \left( {\frac{2}{r}\left( {\frac{\partial u}{{\partial r}}} \right)^{3} + 6\left( {\frac{\partial u}{{\partial r}}} \right)^{2} \frac{{\partial^{2} u}}{{\partial r^{2} }}} \right) - \delta_{hnf} B_{o}^{2} u,$$

3 $$\begin{gathered} \left( {\rho Cp} \right)_{hnf} \left( {v\frac{\partial T}{{\partial r}} + u\frac{\partial T}{{\partial x}}} \right) = k_{hnf} \left( {\frac{{\partial^{2} T}}{{\partial r^{2} }} + \frac{1}{r}\frac{\partial T}{{\partial r}}} \right) + 2\beta_{3} \left( {\frac{\partial u}{{\partial r}}} \right)^{4} + \mu_{hnf} \left( {\frac{\partial u}{{\partial r}}} \right)^{2} \hfill \\ + Q_{o} \left( {T_{w} - T_{\infty } } \right) - \frac{1}{r}\frac{\partial }{\partial r}\left( {rq_{r} } \right), \hfill \\ \end{gathered}$$

with the suitable no-slip boundary conditions (BCs) offered as^10,24^:

4 $$\left. \begin{gathered} v = 0,\,\,u = u_{w} ,\,\,\,T = T_{w} ,\,\,\,\,\,\,\,\,\,\,\,\,\,\,\,\,{\mathrm{at}}\,\,\,\,r = R\left( x \right), \hfill \\ u \to u_{\infty } ,\,\,\,T \to 0,\,\,\,\,\,\,\,\,\,\,\,\,\,\,\,\,\,\,\,\,\,\,\,\,\,\,\,{\mathrm{as}}\,\,\,\,r \to \infty . \hfill \\ \end{gathered} \right\}$$

The following boundary conditions apply to the governing equations (1)–(3) and are obtained from the flow problem’s physical constraints. Here, $r = R\left( x \right)$ is the needle surface. It is assumed that the fluid velocity in relation to the boundary is zero when the no-slip condition is applied. This means that the needle’s surface velocity $u_{w}$ is equal to the axial velocity $u.$ The needle must have a radial velocity component *v* of zero since it is solid and impermeable. As the thermal boundary condition, the needle surface is kept at a steady temperature $T_{w} .$ The axial velocity $u$ approaches the free-stream velocity $u_{\infty }$ since it is believed that the needle has not affected the flow. $T$ asymptotically gets closer to $T_{\infty }$, the ambient temperature. In Eqs. (2)–(3), the notations of viscosity $\left( {\mu_{hnf} } \right),$ Heat capacity $\left( {\left( {Cp} \right)_{hnf} } \right),$ density $\left( {\rho_{hnf} } \right),$ thermal conductivity $\left( {k_{hnf} } \right),$ and electrical conductivity $\left( {\delta_{hnf} } \right),$ are the HNF.

Now presenting the similarity transformations^24^,

5 $$\eta = \frac{{Ur^{2} }}{{\nu_{f} x}},\;\;\psi = \nu_{f} xf\left( \eta \right)\,\;{\mathrm{and}}\,\;\theta \left( \eta \right) = \frac{{T - T_{\infty } }}{{T_{w} - T_{\infty } }},$$

where the continuity equation is inherently satisfied by introducing a stream function $\psi$ with $v = - \frac{1}{r}\frac{\partial \psi }{{\partial x}}\,\,\,{\mathrm{and}}\,\,\,u = \frac{1}{r}\frac{\partial \psi }{{\partial r}}\,,$ and the governing PDEs are reduced to ODEs.

6 $$\frac{{\mu_{r} }}{{\rho_{r} }}\left( {2\eta f^{\prime\prime\prime} + 2f^{\prime\prime}} \right) + ff^{\prime\prime} - \frac{{\delta_{r} }}{{\rho_{r} }}Mf^{\prime} + \frac{{\beta {\mathrm{Re}} \eta }}{{2\rho_{r} }}\left( {\left( {f^{\prime\prime}} \right)^{3} + 3\eta \left( {f^{\prime\prime}} \right)^{2} f^{\prime\prime\prime}} \right) = 0,$$

7 $$\begin{gathered} 2k_{r} \left( {\eta \theta^{\prime\prime} + \theta^{\prime}} \right) + Nr\left( {\eta \theta^{\prime\prime} + \frac{{\theta^{\prime}}}{2}} \right)\left( {1 + \left( {\theta_{w} - 1} \right)\theta } \right)^{3} + 3\eta Nr\left( {\theta^{\prime}} \right)^{2} \left( {\theta_{w} - 1} \right)\left( {\left( {\left( {\theta_{w} - 1} \right)\theta + 1} \right)^{2} } \right) + \Pr H\theta \hfill \\ + \eta Ec\Pr \mu_{r} \left( {f^{\prime\prime}} \right)^{2} + \Pr \left( {\rho Cp} \right)_{r} f\theta^{\prime} + \eta^{2} \beta {\mathrm{Re}} Ec\Pr \left( {f^{\prime\prime}} \right)^{4} = 0, \hfill \\ \end{gathered}$$

with BCs becomes,

8 $$\left. \begin{gathered} f^{\prime}\left( \eta \right) = \frac{a}{2},\,\,f\left( \eta \right) = \frac{\chi a}{2},\,\,\,\theta \left( \eta \right) = 1,\,\,\,\,\,{\mathrm{at}}\,\,\,\,\,\,\eta \to \chi ,\,\, \hfill \\ f^{\prime}\left( \eta \right) = \frac{1 - a}{2},\,\theta \left( \eta \right) = 0,\,\,\,\,\,\,\,\,\,\,\,\,\,\,\,\,\,\,\,\,\,\,\,\,\,\,{\mathrm{as}}\,\,\,\,\,\,\eta \to \infty . \hfill \\ \end{gathered} \right\}$$

Furthermore, assume $$\chi = \eta$$ that represents the needle thickness or size, as well as $$a = {{u_{w} } \mathord{\left/ {\vphantom {{u_{w} } U}} \right. \kern-0pt} U}$$ the velocity ratio factor and $$\beta = \frac{{256U^{2} \beta_{3} }}{{\rho_{f} \nu_{f} }}$$ the third-grade factor, $${\mathrm{Re}} = \frac{Ux}{{\nu_{f} }}$$ the local Reynolds number, $$M = \frac{{\delta_{f} B_{o}^{2} }}{{2\rho_{f} U}}$$ the magnetic factor, $$\theta_{w} = \frac{{T_{w} }}{{T_{\infty } }}$$ the thermal ratio factor, $$Nr = \frac{{16T_{\infty }^{3} \delta^{**} }}{{k^{**} k_{f} }}$$ the thermal radiation, $$Ec = \frac{{u_{w}^{2} }}{{2\left( {T_{w} - T_{\infty } } \right)\left( {Cp} \right)_{f} }}$$ the Eckert number, $$H = \frac{{Q_{o} }}{{2U\left( {\rho Cp} \right)_{f} }}$$ the heat generation factor, and $$\Pr = \frac{{\mu_{f} \left( {Cp} \right)_{f} }}{{k_{f} }}$$ the Prandtl number.

Eq. 9 shows the thermophysical characteristics of HNF^20,21,45,47,51^.

9 $$\begin{gathered} \frac{{\rho_{hnf} }}{{\rho_{f} }} = \rho_{r} = \left[ {\left( {1 - \phi_{2} } \right)\left\{ {\left( {1 - \phi_{1} } \right) + \frac{{\phi_{1} }}{{\rho_{f} }}\rho_{{s_{1} }} } \right\} + \frac{{\phi_{2} }}{{\rho_{f} }}\rho_{{s_{2} }} } \right],\;\frac{{\mu_{hnf} }}{{\mu_{f} }} = \mu_{r} = \frac{1}{{\left( {1 - \phi_{1} } \right)^{2.5} \left( {1 - \phi_{2} } \right)^{2.5} }}, \hfill \\ \frac{{\left( {\rho C_{\rho } } \right)_{hnf} }}{{\left( {\rho C_{\rho } } \right)_{f} }} = \left( {\rho C_{\rho } } \right)_{r} = \frac{{\phi_{2} \left( {\rho C_{\rho } } \right)_{{s_{2} }} }}{{\left( {\rho C_{\rho } } \right)_{f} }} + \left( {1 - \phi_{2} } \right)\left[ {\left( {1 - \phi_{1} } \right) + \frac{{\phi_{1} \left( {\rho C_{\rho } } \right)_{{s_{1} }} }}{{\left( {\rho C_{\rho } } \right)_{f} }}} \right],\,\;\alpha_{r} = \frac{{k_{r} }}{{\left( {\rho C_{P} } \right)_{r} }}, \hfill \\ \end{gathered}$$

$$\frac{{k_{r} }}{{k_{nf} }} = \frac{{2k_{nf} - 2\phi_{1} \left( {k_{{s_{1} }} - k_{nf} } \right) + k_{{s_{1} }} }}{{2k_{nf} + \phi_{1} \left( {k_{{s_{1} }} - k_{nf} } \right) + k_{{s_{1} }} }},\;\frac{{k_{nf} }}{{k_{f} }} = \frac{{2k_{f} - 2\phi_{2} \left( {k_{{s_{2} }} - k_{f} } \right) + k_{{s_{2} }} }}{{2k_{f} + \phi_{2} \left( {k_{{s_{2} }} - k_{f} } \right) + k_{{s_{2} }} }},\,\,\,k_{r} = \frac{{k_{hnf} }}{{k_{f} }},\;\;\delta_{r} = \frac{{\delta_{hnf} }}{{\delta_{f} }},$$

$$\sigma_{r} = \left[ {\frac{{\left( {1 + 2\phi_{2} } \right)\sigma_{{s_{2} }} + 2\sigma_{bf} \left( {1 - \phi_{2} } \right)}}{{\left( {1 - \phi_{2} } \right)\sigma_{{s_{2} }} + \sigma_{bf} \left( {2 + \phi_{2} } \right)}}} \right]\sigma_{bf} ,\;\;\sigma_{bf} = \left[ {\frac{{\sigma_{{s_{1} }} \left( {1 + 2\phi_{1} } \right) + 2\sigma_{f} \left( {1 - \phi_{1} } \right)}}{{\sigma_{{s_{1} }} \left( {1 - \phi_{1} } \right) + \sigma_{f} \left( {2 + \phi_{1} } \right)}}} \right]\sigma_{f} ,$$

where ${\mathrm{Al}}_{{2}} {\mathrm{O}}_{{3}}$ and ${\mathrm{TiO}}_{{2}}$ are nanosized materials having the volume fractions $\phi_{1}$ and $\phi_{2}$ respectively. $\alpha_{r}$ thermal diffusivity of HNF. Also $s_{1}$ and $s_{2}$ denote the ${\mathrm{Al}}_{{2}} {\mathrm{O}}_{{3}}$ and ${\mathrm{TiO}}_{{2}}$ solid nanosized materials, respectively. Table 1 shows the thermophysical characteristics of Sodium Alginate (SA) and nanomaterials.

**Table 1:** The thermophysical characteristics of ${\mathrm{Al}}_{{2}} {\mathrm{O}}_{{3}}$, as well as ${\mathrm{TiO}}_{{2}}$ and SA^21,41,45^.

Physical properties	$\rho \left( {kg/m^{3} } \right)$	$Cp\left( {J/kgK} \right)$	$k\left( {W/mK} \right)$	$\delta \left( {\Omega /m} \right)$
*SA*	989	4175	0.6376	$2.6 \times 10^{ - 4}$
${\mathrm{Al}}_{{2}} {\mathrm{O}}_{{3}}$	3970	765	40	$3.69 \times 10^{7}$
${\mathrm{TiO}}_{{2}}$	4250	686.20	8.9538	$0.9 \times 10^{5}$

### Engineering quantities

The surface drag force and thermal energy transmission rate are recognized as

10 $$C_{f} = \frac{{\tau_{w} }}{{\rho_{f} U^{2} }},\;{\mathrm{and}}\;Nu_{x} = \frac{{xq_{w} }}{{k_{f} \left( {T_{w} - T_{\infty } } \right)}},$$

where $$\tau_{w} = \frac{\partial u}{{\partial r}}\left( {\mu_{hnf} + 2\beta_{3} \left( {\frac{\partial u}{{\partial r}}} \right)^{2} } \right)_{r = \chi }$$ and $$q_{w} = \frac{\partial T}{{\partial r}}\left( { - k_{hnf} - \frac{{16\delta^{**} T^{3} }}{{3k^{**} }}} \right)_{r = \chi }$$ are known as the shear stress and heat flux, respectively.

Utilizing (5), we get

11 $$\left. \begin{gathered} \left( {\mathrm{Re}} \right)^{0.5} C_{f} = 8\sqrt \chi \left( {\mu_{r} + \beta R\left( {f^{\prime\prime}\left( \chi \right)} \right)^{2} } \right)f^{\prime\prime}\left( \chi \right), \hfill \\ \left( {\mathrm{Re}} \right)^{ - 0.5} Nu_{x} = - 2\sqrt \chi \theta^{\prime}\left( \chi \right)\left[ {k_{r} + Nr\left( {1 + \theta \left( \chi \right)\left( {\theta_{w} - 1} \right)} \right)^{3} } \right]. \hfill \\ \end{gathered} \right\}$$

## Fundamental concepts of fuzzy analysis, TFN and FDE

This section deals with the background concepts and information required to understand the current work better^61–68^.

### Definition 3.1.

Fuzzy set: Let us consider a universal set *G*. A fuzzy set, say, $\overline{F}$ is defined as the collection of ordered pairs $\left( {\varepsilon ,\,\,m\left( \varepsilon \right)} \right),$ where $\varepsilon \in G$ and $m\left( \varepsilon \right)$ denote the membership value of $\varepsilon \in G.$ It may be expressed as $\overline{F} = \left\{ {\left( {\varepsilon ,\,m\left( \varepsilon \right)} \right):\varepsilon \in G,\,m\left( \varepsilon \right) \in \left[ {0,\,1} \right]} \right\},$ the membership function $m\left( \varepsilon \right)$ has a range of [0,1].

### Definition 3.2.

**Fig. 2:**
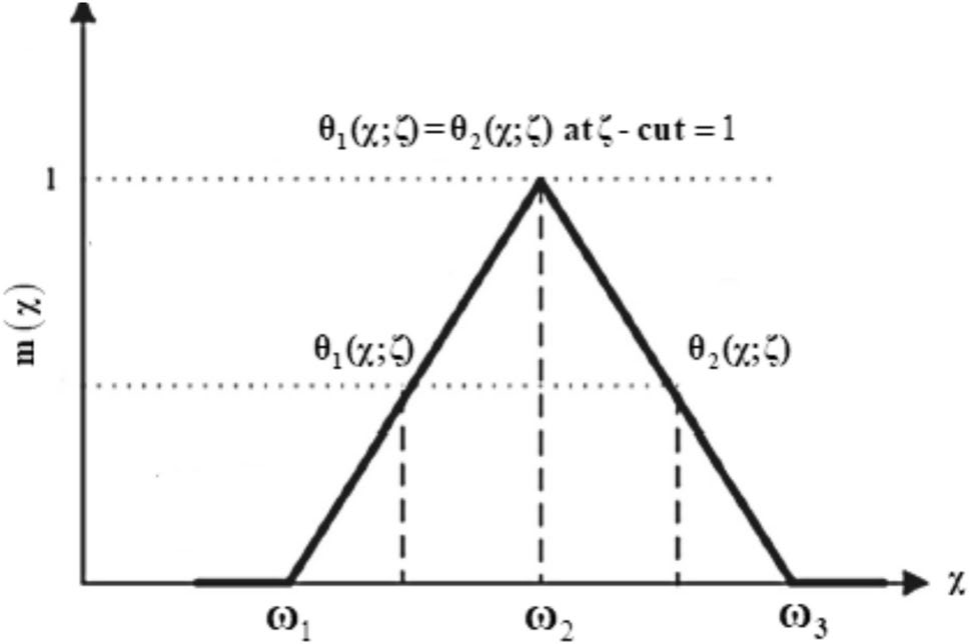
Membership function of a symmetrical TFN.

Triangular fuzzy number (TFN): TFNs are commonly employed in the modeling of uncertain structural factors due to their computational simplicity and ease of interpretation. A TFN $X = \left[ { \, \omega_{1} ,\omega_{2} ,\omega_{3} } \right]$ is fully characterized by three values: $\omega_{1}$ (lower value), $\omega_{2}$ (most belief value), and $\omega_{3}$ (upper value), as illustrated in Fig. 2. Corresponding to this representation, the membership function $m\left( \varepsilon \right)$ of the TFN can be defined as:

12 $$m(\chi ) = \left\{ \begin{gathered} \frac{{\omega_{1} - \varepsilon }}{{\omega_{2} - \omega_{1} }}\,\,\,\,\,{\mathrm{for}}\,\,\,\,\varepsilon \in [\omega_{1} ,\,\,\omega_{2} ], \hfill \\ \,\frac{{\varepsilon - \omega_{3} }}{{\omega_{3} - \omega_{2} }}\,\,\,\,{\mathrm{for}}\,\,\,\,\varepsilon \in [\omega_{2} ,\,\,\omega_{3} ], \hfill \\ \,\,\,\,\,\,0,\,\,\,\,\,\,\,\,\,\,\,\,\,\,\,\,{\mathrm{otherwise}}{.} \hfill \\ \end{gathered} \right.$$

### Definition 3.3.

The $\zeta {\text{ - cut}}$ technique: The $\zeta {\text{ - cut}}$ technique is a fundamental method for translating a fuzzy set into a series of crisp intervals, enabling numerical computation. The TFNs are transformed into interval numbers through $\zeta {\text{ - cut}}$ approach, which is written as $\overline{\theta }\left( {\chi ,\,\zeta } \right) = \left[ {\theta_{1} (\chi ;\,\zeta ),\theta_{2} (\chi ;\,\zeta )} \right] = \left[ {\omega_{1} + \zeta (\omega_{2} - \omega_{1} ),\,\,\omega_{3} - \zeta (\omega_{3} - \omega_{2} )} \right],$ where 

To handle such TFNs, use FDEs with the help of the $\zeta {\text{ - cut}}$ technique. The FDEs are converted into lower and upper bounds of FDEs like $\theta_{1} (\chi ;\,\zeta )\,\,and\,\,\theta_{2} (\chi ;\,\zeta )$ respectively.

### Definition 3.4.

Fuzzy process: Let *I* will be a real interval. A mapping $\tilde{u}:I \to P$ is called a fuzzy process, defined as $\overline{\theta }(\chi ;\,\zeta ) = \left[ {\theta_{1} (\chi ;\,\zeta ),\theta_{2} (\chi ;\,\zeta )} \right],\,\,\chi \in I\,$ and $\zeta \in [0,\,1].$ The derivative $\frac{{d\overline{\theta }(\chi ;\,\zeta )}}{d\chi } \in P$ of a fuzzy process $\overline{\theta }(\chi ;\,\zeta )$ is defined by”

13 $$\frac{{d\overline{\theta }(\chi ;\,\zeta )}}{d\chi } = \left[ {\frac{{d\overline{\theta }_{1} (\chi ;\,\zeta )}}{d\chi },\,\frac{{d\overline{\theta }_{2} (\chi ;\,\zeta )}}{d\chi }} \right].$$

The basic strength of the fuzzy framework relative to deterministic modeling is that it can quantify and describe the uncertainty of the output. A deterministic model gives a single solution point, which, although convenient for a particular input, says nothing about the sensitivity of the solution to parameter changes. Conversely, the fuzzy model yields a fuzzy output profile, which is often graphically presented as a “spread” or range of values, that provides deep insights. In addition, it is more appropriate than a probabilistic model for this use because it avoids the need to use large statistical data to define specific probability distribution information that tends to be lacking for new hybrid nanofluids. Instead, it requires only an interval of credible probability and a most probable value, which is useful in early-stage analysis where numerical quantification of uncertainty from sparse data is paramount for good design.

The TFN defines a specific form of FN by assigning a degree of membership to each $\zeta {\text{ - cut}}$ within a defined interval. TFNs are characterized by triangular MFs that vary continuously from 0 to 1, as illustrated in Fig. 2. The triangular structure is formed by a left-side monotonically non-decreasing function and a right-side monotonically non-increasing function, converging at the peak (most plausible value). This formulation allows for a simple yet effective representation of uncertainty. In the present study, TFNs are employed to model imprecise nanoparticle volume fractions, facilitating a comparative and uncertainty-based analysis between conventional NFs and HNFs.

The crisp values $\phi_{1}$ and $\phi_{2}$ are said to be TFNs controlled by $\zeta {\text{ - cut}}\,\left( {0 \le \zeta \le 1} \right)$ is presented in Table 2. The triangular MFs, which range from 0 to 1, are explained using TFNs. The said problem is developed using the investigated range.

**Table 2:** TFNs of the volume fraction of fuzzy nanosized materials^61–68^.

Fuzzy numbers	Crisp value	TFN	$\zeta {\text{ - cut}}$
$$\phi_{1}$$$$\left( {{\mathrm{Al}}_{{2}} {\mathrm{O}}_{{3}} } \right)$$	[1 $$\times {10}^{-2}-4\times {10}^{-2}$$]	[0, 0.1, 0.2]	$$\left[ {0.1\zeta ,\,\,0.2 - 0.1\zeta } \right],\,\,\zeta \in \left[ {0,\,1} \right]$$
$$\phi_{2}$$$$\left( {{\mathrm{Cu}}} \right)$$	[1 $$\times {10}^{-2}-4\times {10}^{-2}$$]	[0, 0.1, 0.2]	$$\left[ {0.1\zeta ,\,\,0.2 - 0.1\zeta } \right],\,\,\zeta \in \left[ {0,\,1} \right]$$

## Results and discussion

**Fig. 3:**
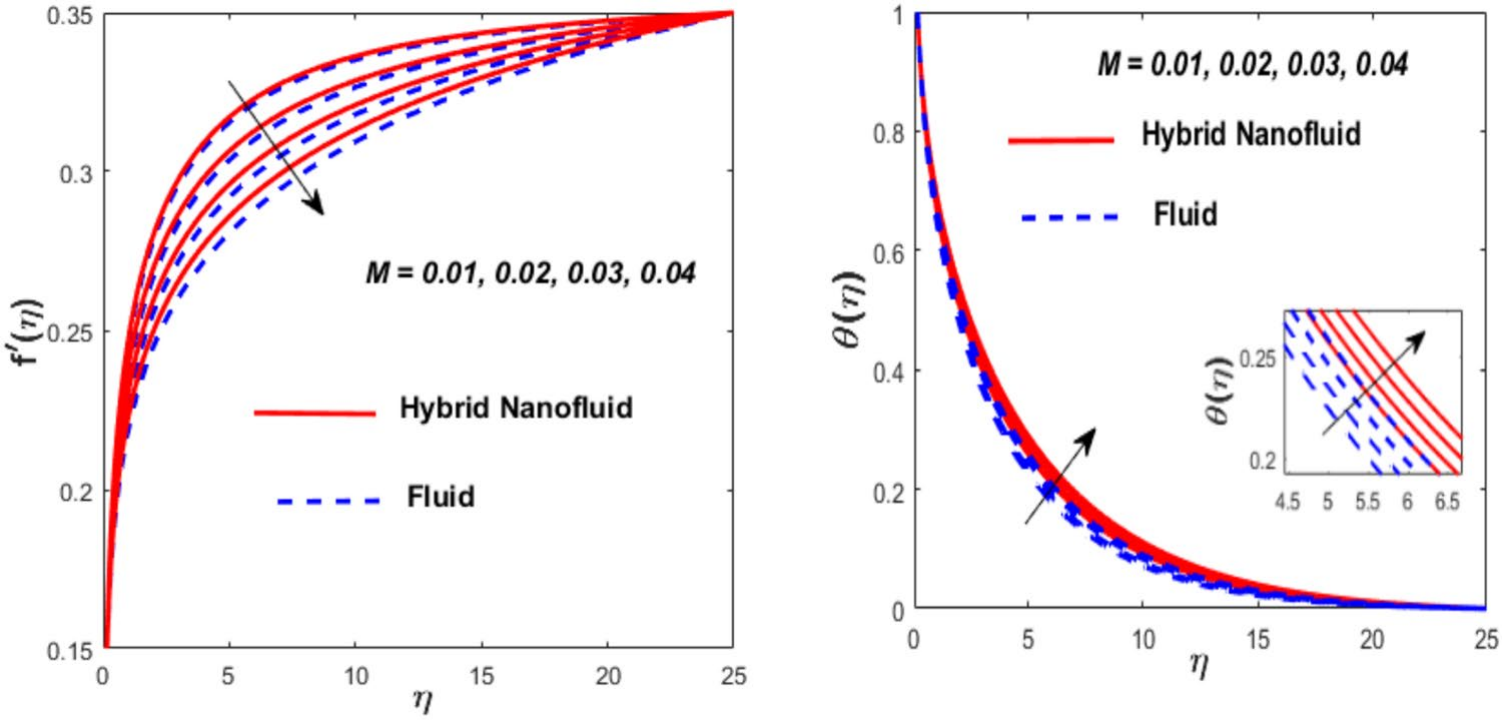
Effect of *M* on $f^{\prime}\left( \eta \right)$ and $\theta \left( \eta \right).$

**Fig. 4:**
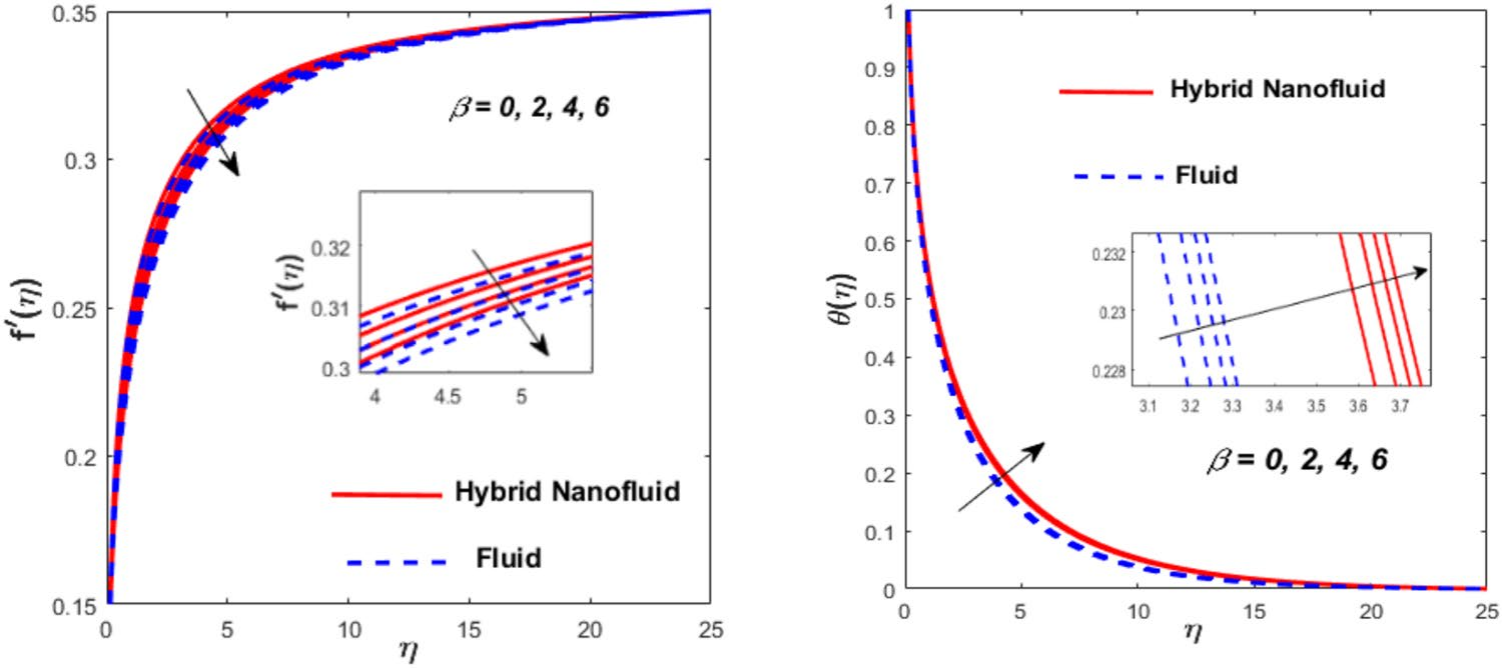
Effect of $\beta$ on $f^{\prime}\left( \eta \right)$ and $\theta \left( \eta \right).$

**Fig. 5:**
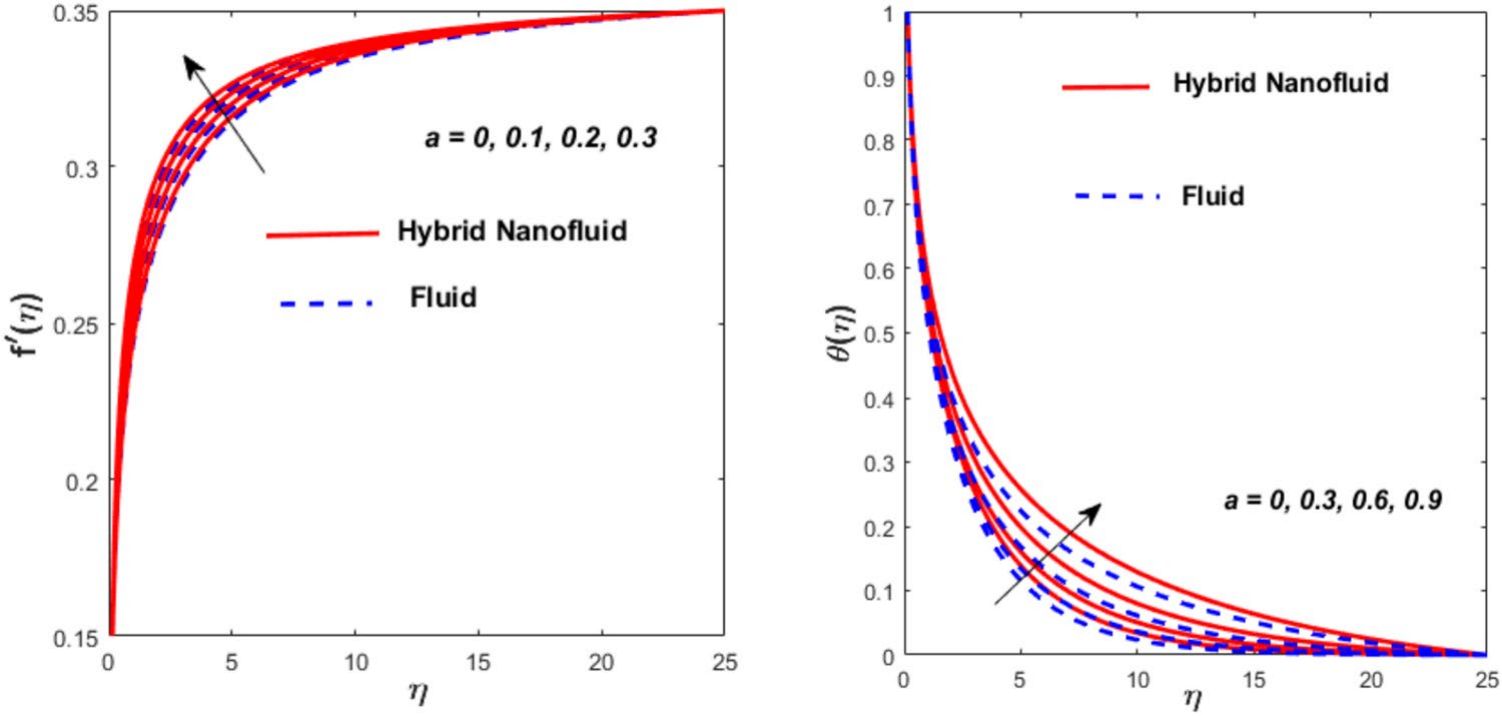
Effect of $a$ on $f^{\prime}\left( \eta \right)$ and $\theta \left( \eta \right).$

**Fig. 6:**
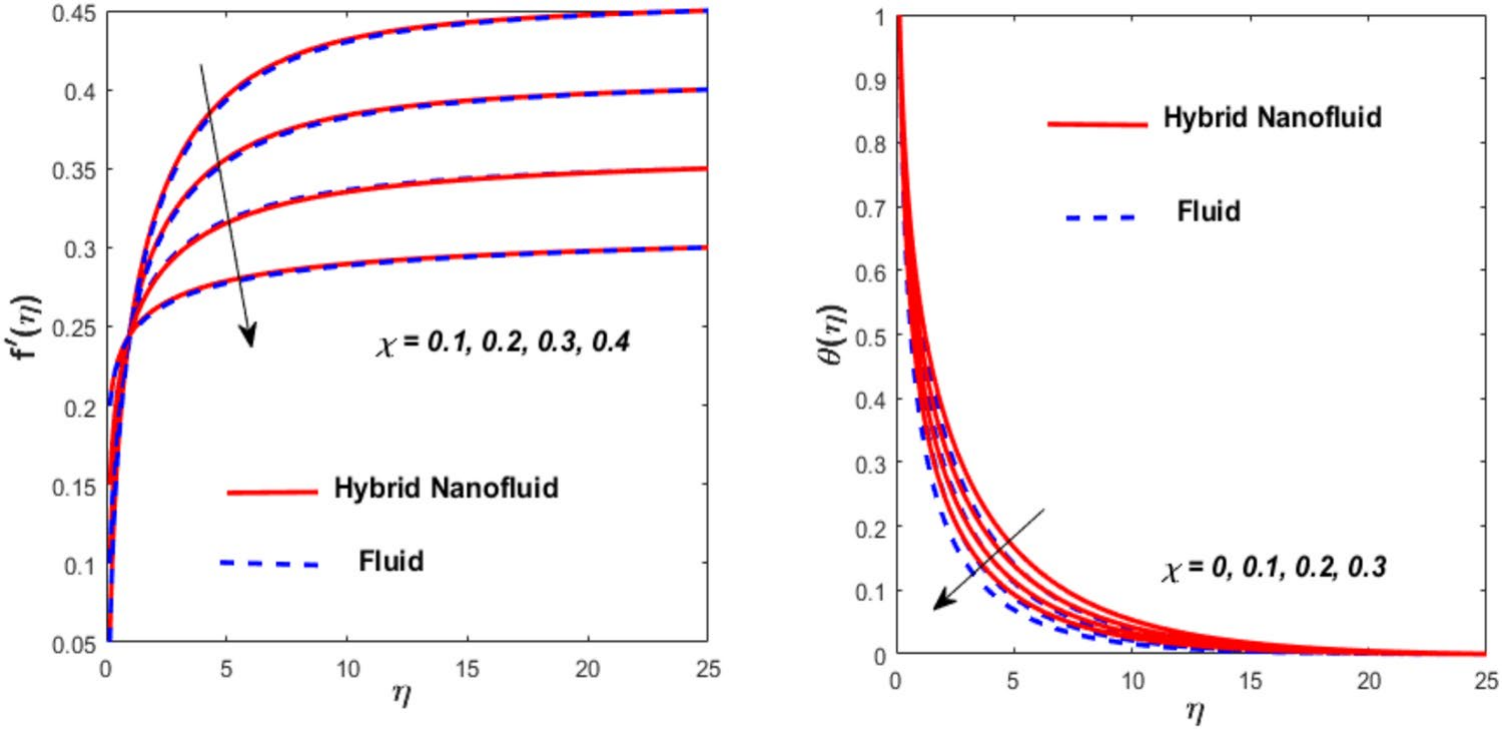
Effect of $\chi$ on $f^{\prime}\left( \eta \right)$ and $\theta \left( \eta \right).$

**Fig. 7:**
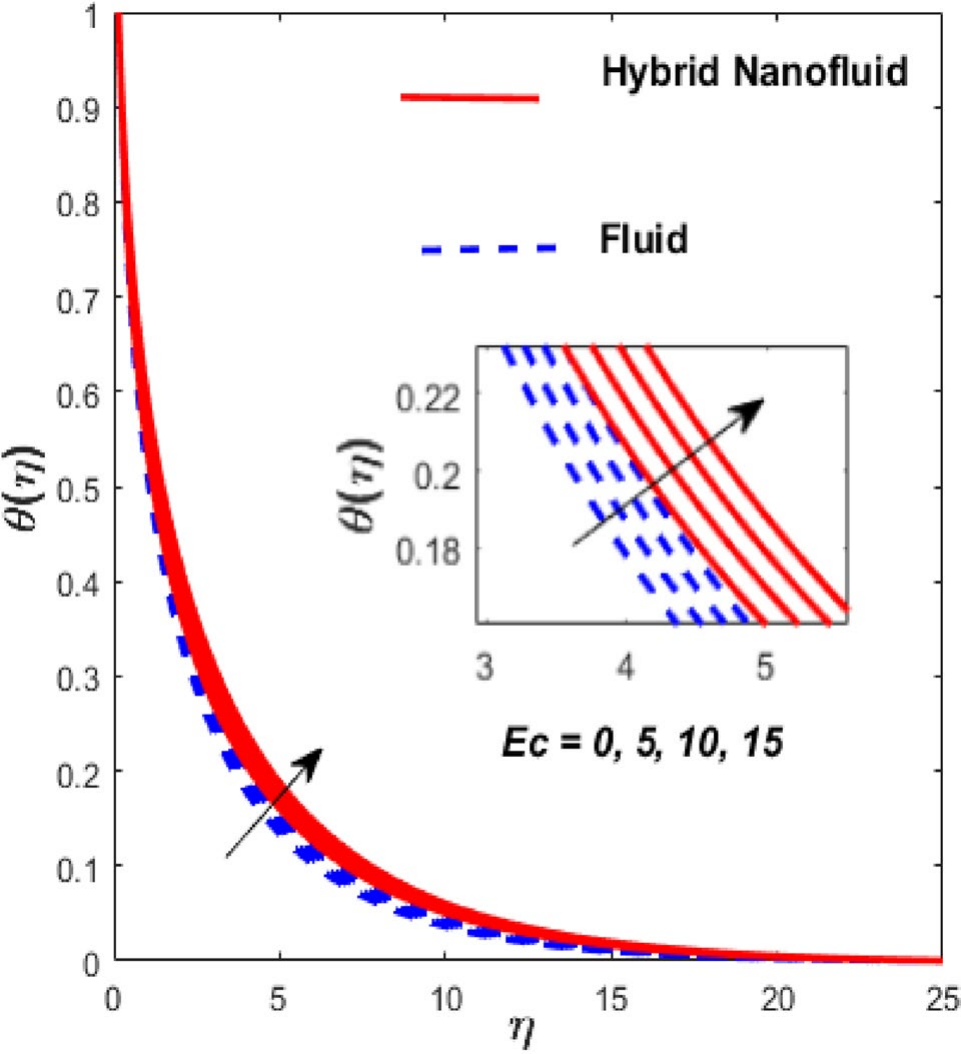
Effect of $Ec$ on $\theta \left( \eta \right).$

**Fig. 8:**
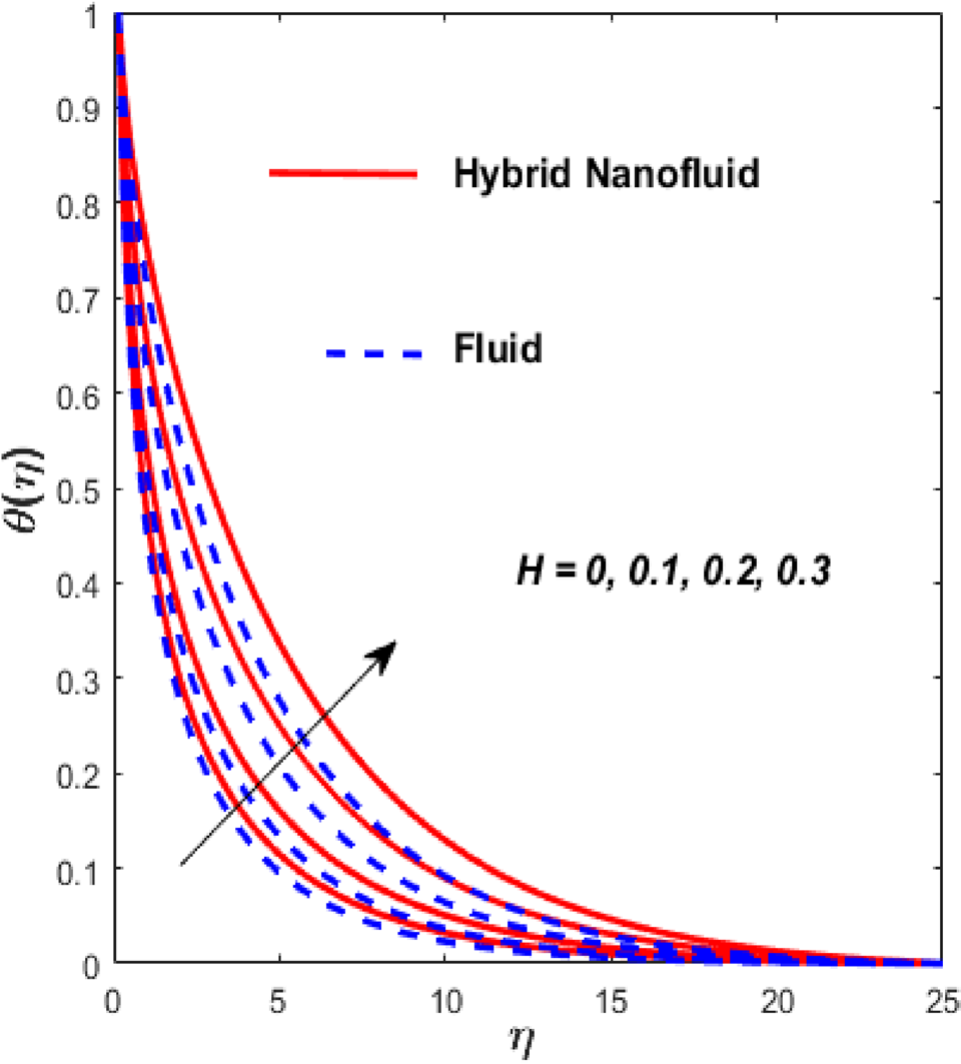
Effect of $H$ on $\theta \left( \eta \right).$

**Fig. 9:**
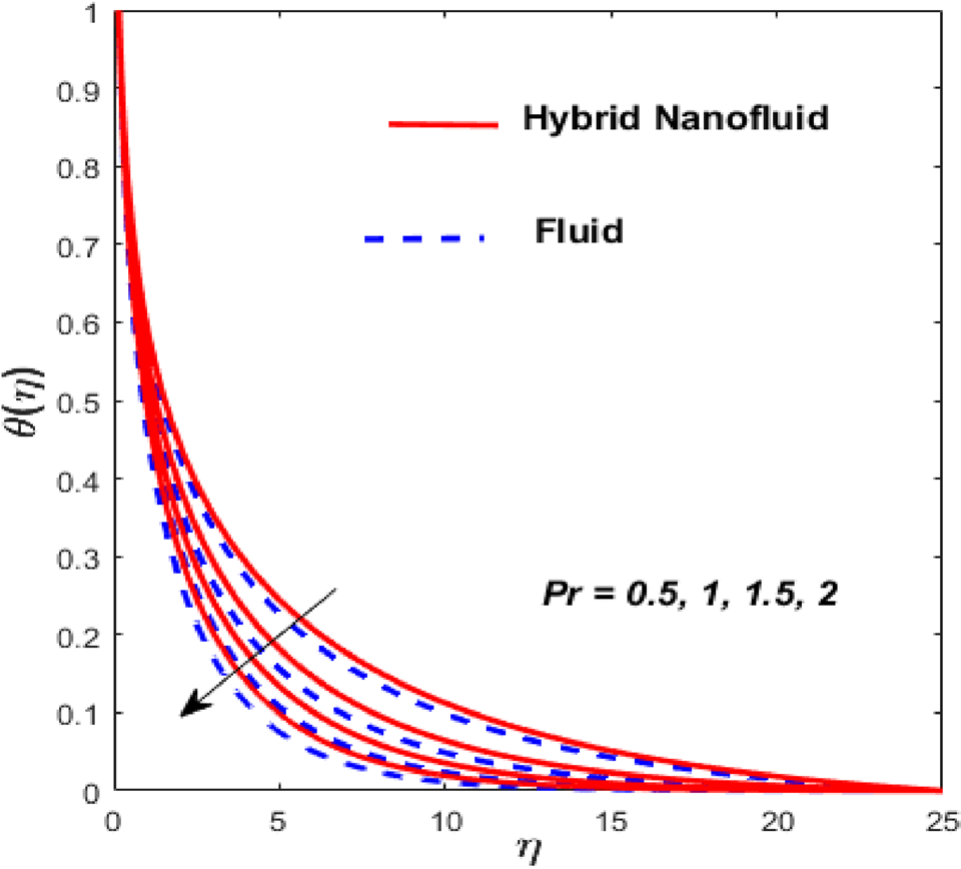
Effect of $\Pr$ on $\theta \left( \eta \right).$

**Fig. 10:**
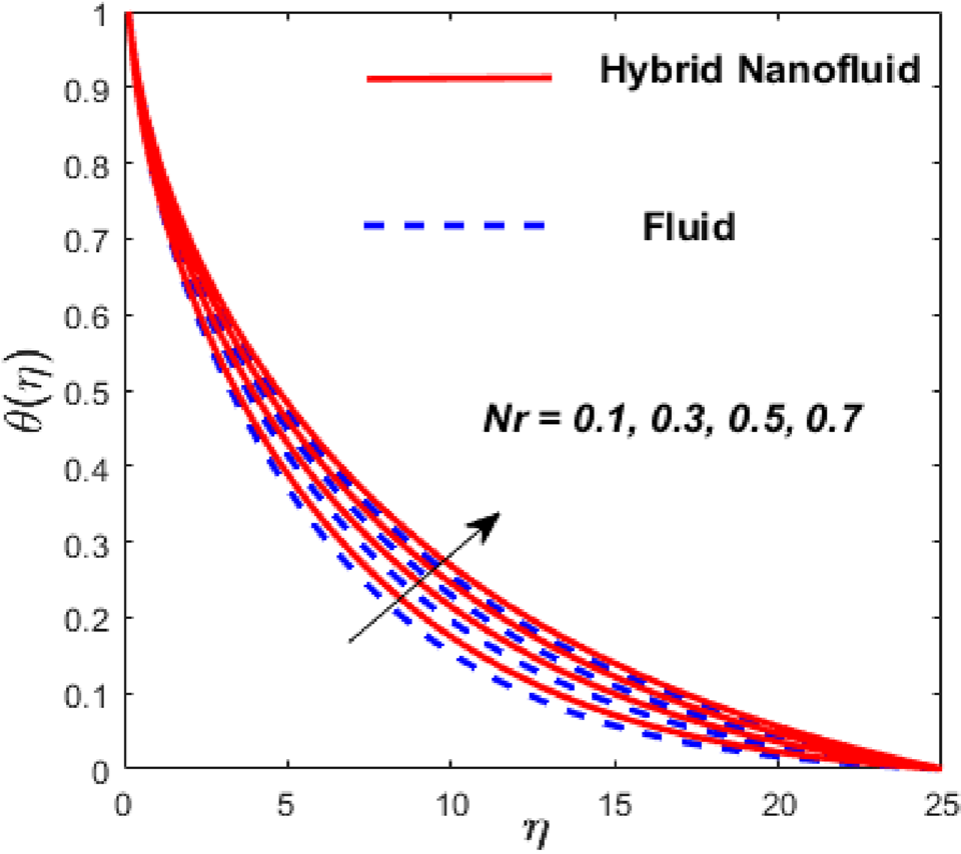
Effect of $Nr$ on $\theta \left( \eta \right).$

**Fig. 11:**
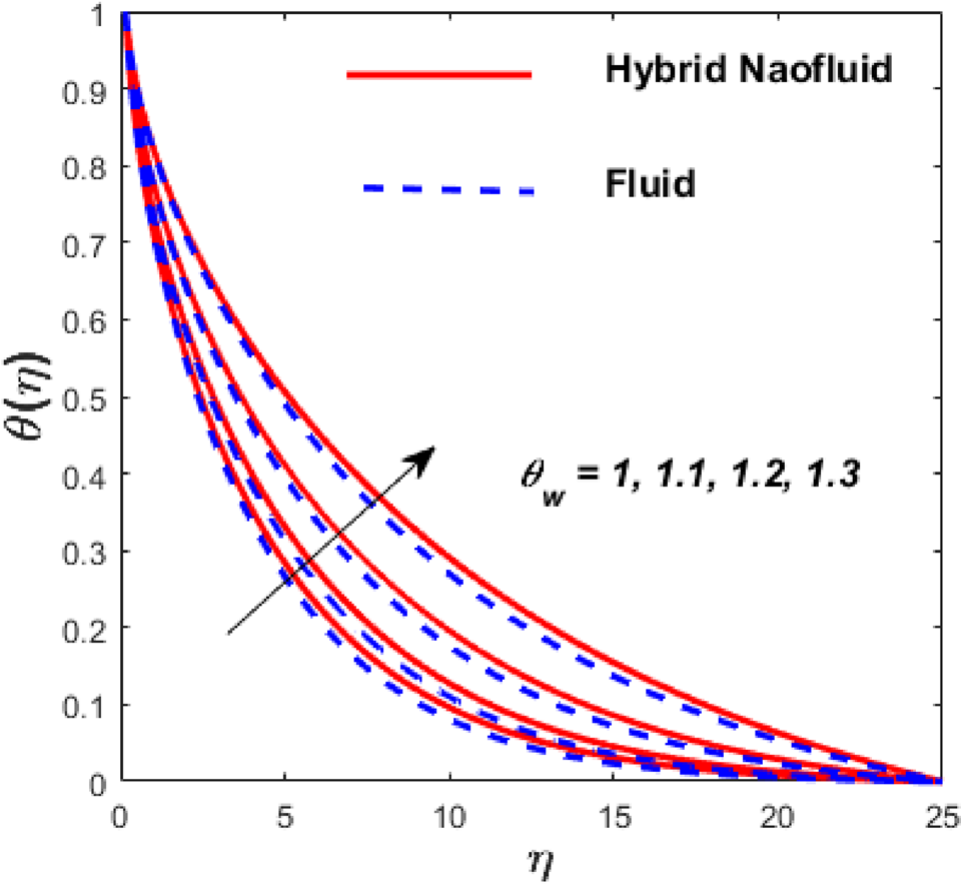
Effect of $\theta_{w}$ on $\theta \left( \eta \right).$

**Fig. 12:**
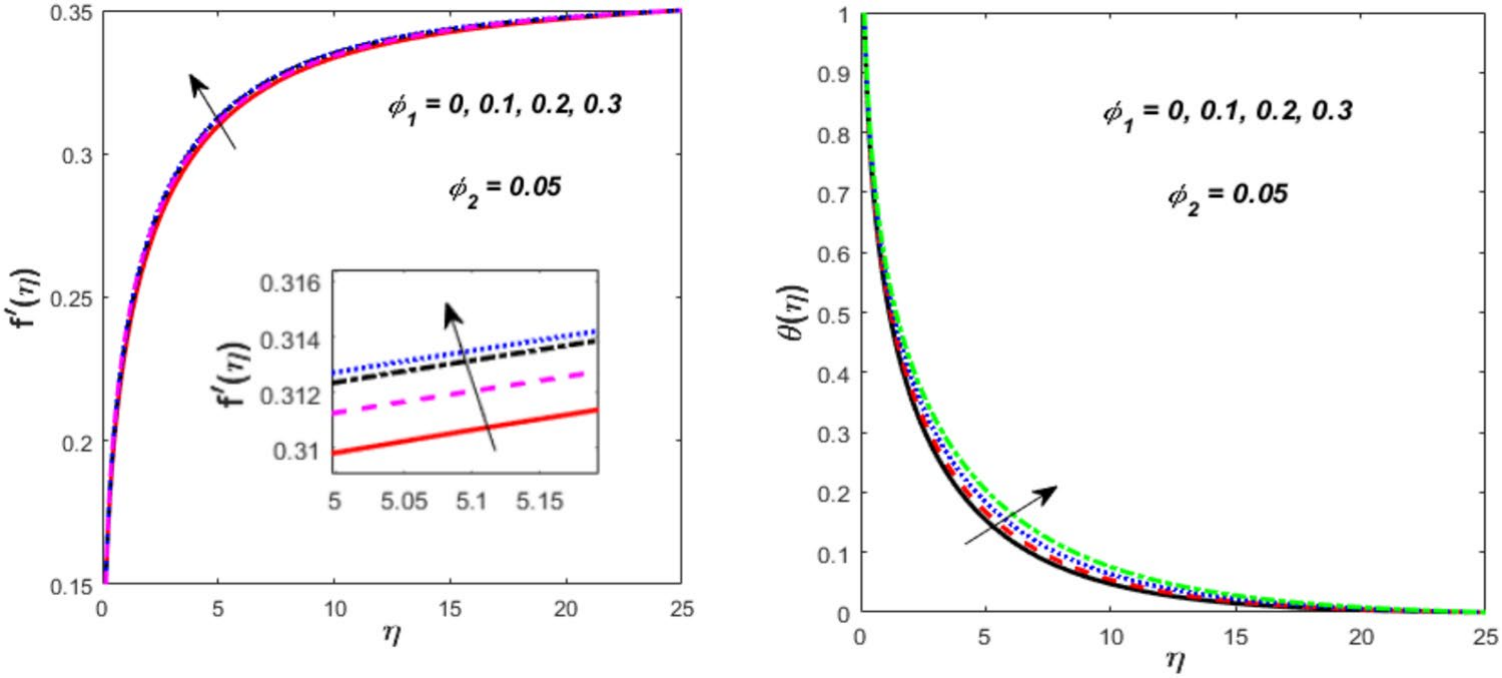
Effect of $\phi_{1}$ on $f^{\prime}\left( \eta \right)$ and $\theta \left( \eta \right).$

**Fig. 13:**
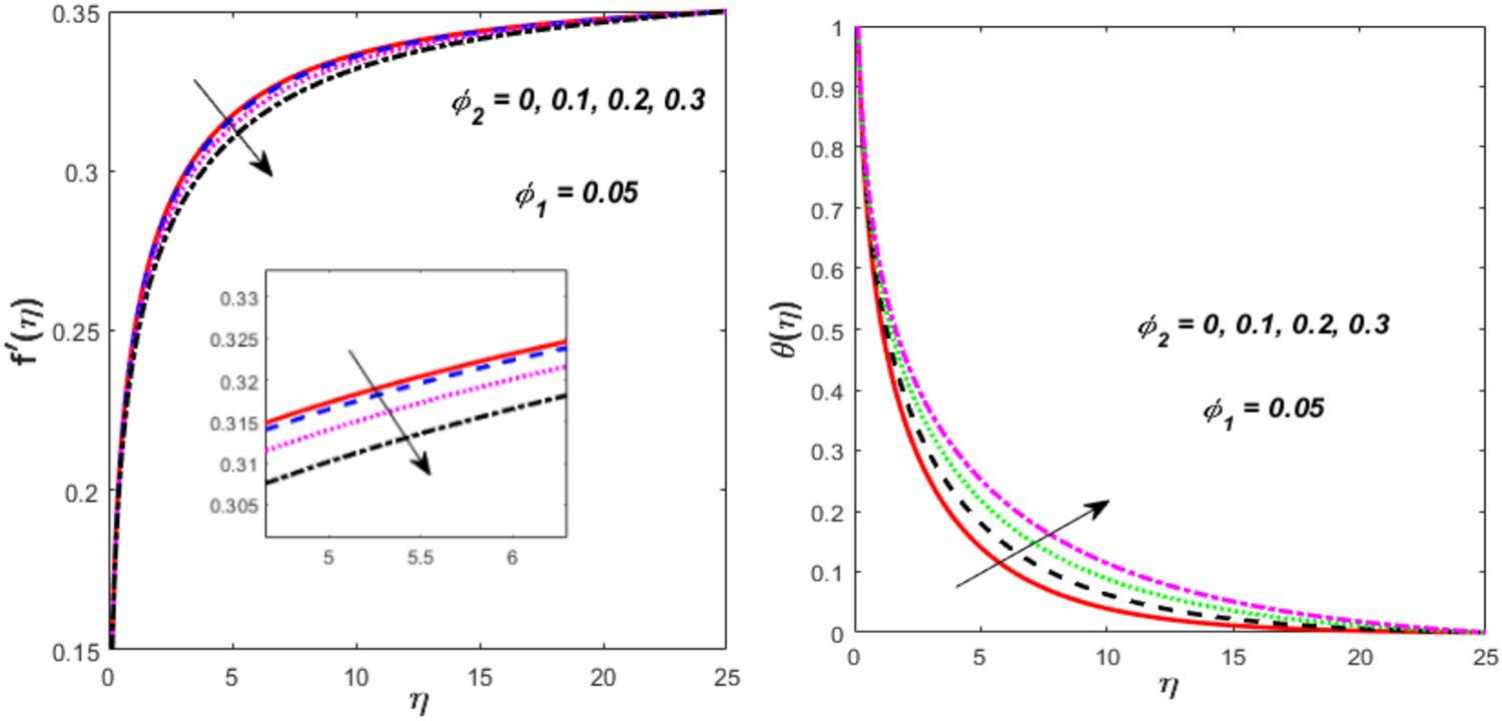
Effect of $\phi_{2}$ on $f^{\prime}\left( \eta \right)$ and $\theta \left( \eta \right).$

**Fig. 14:**
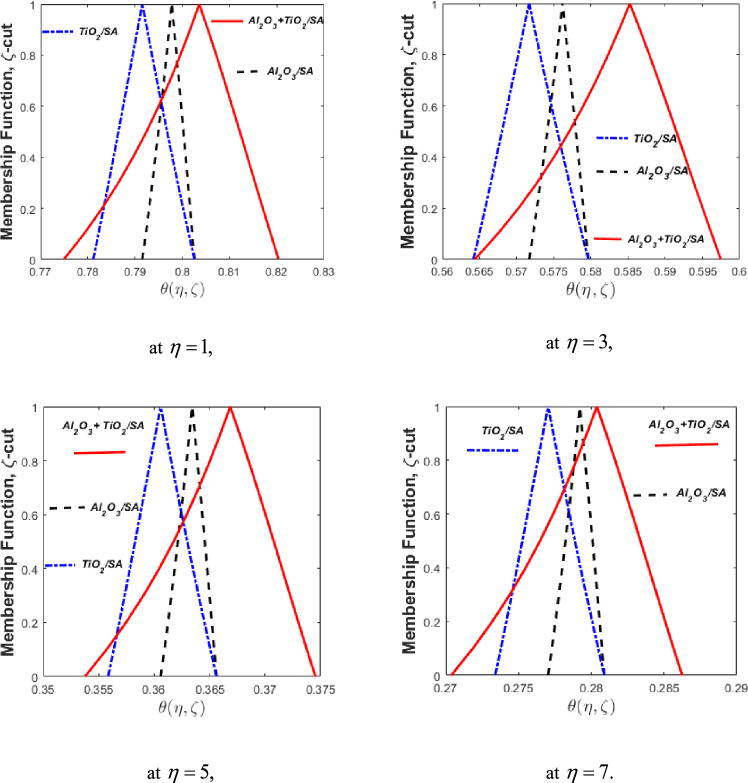
Fuzzy triangular membership plots of the thermal gradient for NFs and HNF when $\phi_{1}$ and $\phi_{2}$ are TFNs.

**Fig. 15:**
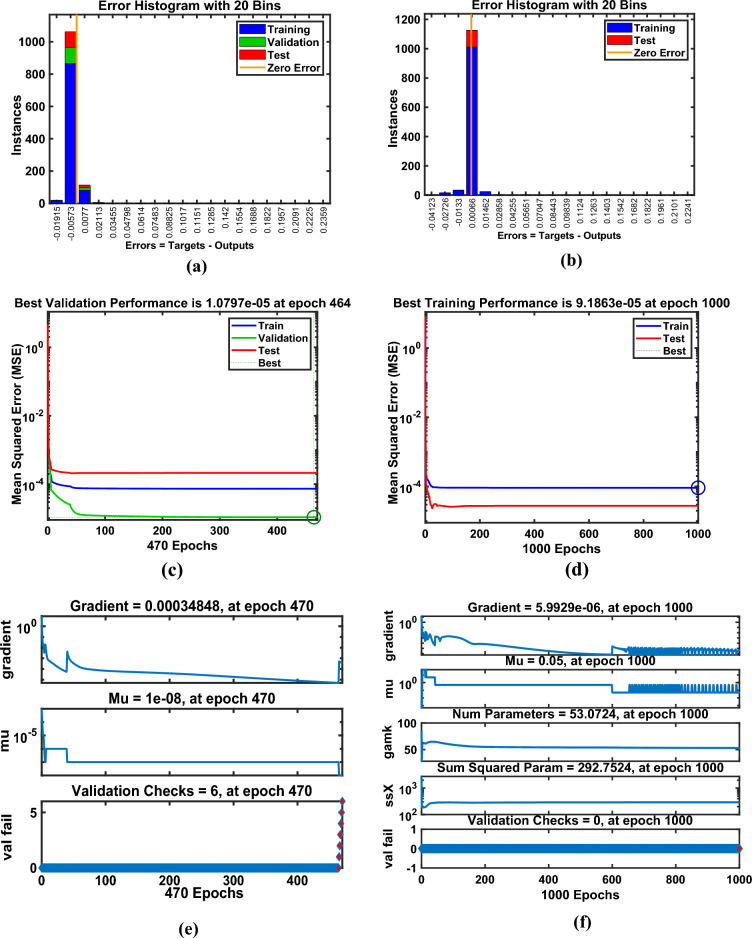

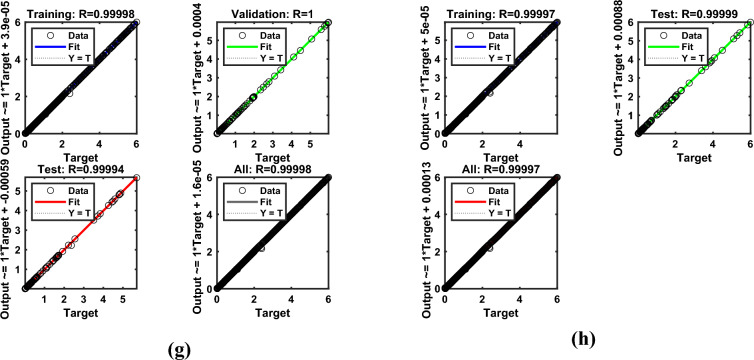
Graphical illustration of Skin friction for LMS (**a, c, e, g**) and for BRS (**b, d, f, h**). (**a, b**) Error Histogram plots, (**c, d**) Performance plots, (**e, f**) Training stat plots, and (**g, h**) Regression plots.

**Fig. 16:**
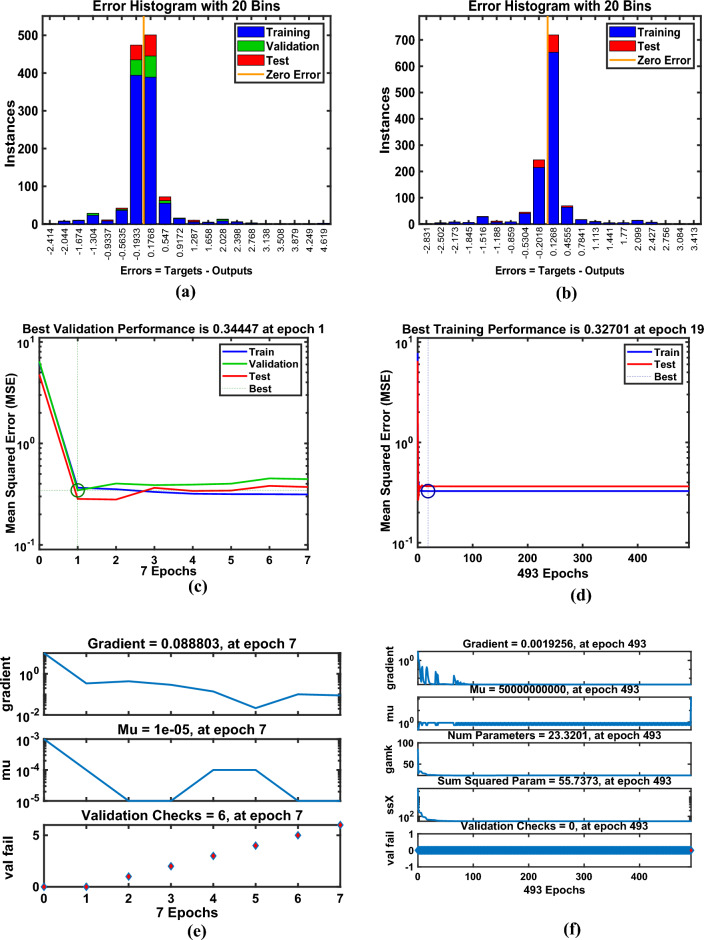

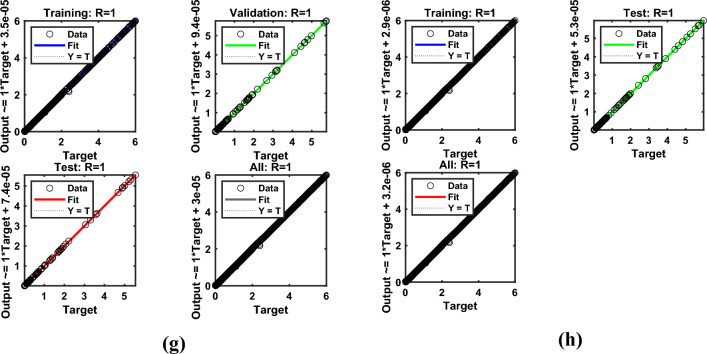
Graphical illustration of Nusselt number for LMS (**a, c, e, g**) and for BRS (**b, d, f, h**). (**a, b**) Error Histogram plots, (**c, d**) Performance plots, (**e, f**) Training stat plots, and (**g, h**) Regression plots.

**Fig. 17:**
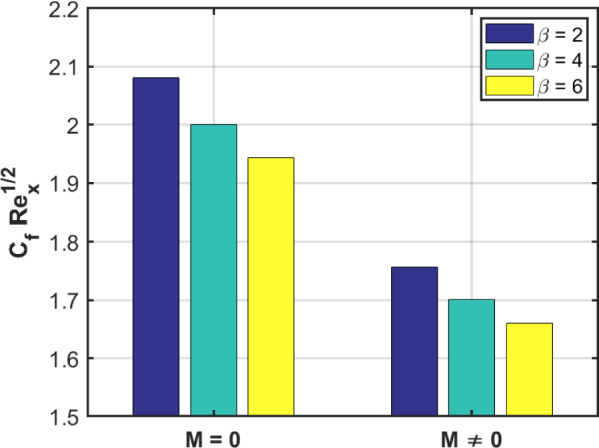
Skin Friction for $M = 0$ and $M \ne 0.$

**Fig. 18:**
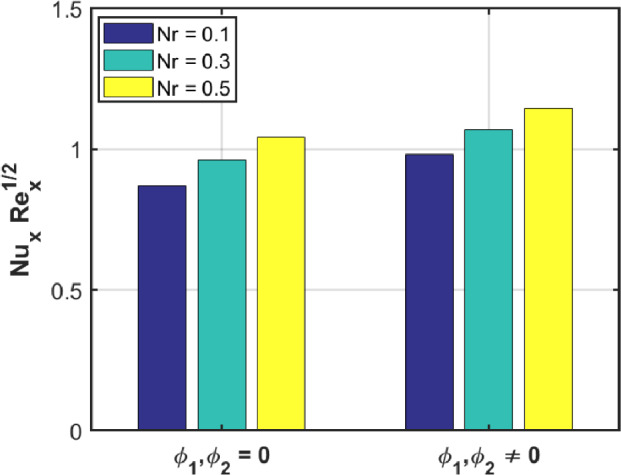
Heat Flux coefficient for $\phi_{1} = \,\phi_{2} = 0$ and $\phi_{1} ,\,\,\phi_{2} \ne 0.$

The ODEs (6) – (7) and the BCs (8) are calculated numerically using the bvp4c algorithm built-in the Matlab software. Different dimensionless factors with standard values, such as $$M = 0.01,$$$$\beta = 0.5,$$ $$\phi_{1} = \phi_{2} = 0.05$$, $$\Pr = 1.2,$$$$Ec = 0.3,$$ $$\theta_{w} = 1.2,$$ $$Nr = 0.3,\,\,Re = 0.5,$$ $$a = 0.3,\,\,\chi = 0.3,$$ and $$H = 0.1$$ effect on velocity and thermal gradients, are illustrated in Figs. (3–18) for liquid (Third-grade) and $$\left( {{\mathrm{Al}}_{{2}} {\mathrm{O}}_{{3}} {\text{ + TiO}}_{{2}} {\mathrm{/SA}}} \right)$$ HNF. Table 3 replicates the performance of numerous factors as discussed above on the Nusselt number as well as the skin friction.

**Table 3:** Comparative investigation for $f^{\prime\prime}\left( \chi \right)$ when $M = Nr = a = \beta = 0 = \phi_{1} = \phi_{2} = 0.$

$\chi$	Ishak et al.^3^	Chen and Smith^2^	Idrees et al.^36^	Song et al.^15^	Aamir Hamid^28^	Present results
0.1	1.28880	1.28881	1.28881	1.28883	1.2888012	1.28507
0.01	8.49240	8.49244	8.49233	8.49129	8.4924121	8.4876
0.001	62.16370	62.16372	62.16370	62.16245	62.163713	62.1591

The results of the present investigation were compared with those from earlier studies by Chen and Smith^2^, Ishak et al.^3^, Song et al.^15^, Aamir^28^, and Idrees^36^, as summarized in Table 3. A strong correlation is observed, confirming the validity of the current model.

Figure 3 illustrates the influence of the magnetic factor $M$ on both velocity and thermal gradients. An increment in $M$ leads to a suppression of the fluid velocity while simultaneously enhancing the thermal gradient. The fluid motion faces a strong barrier from the growing Lorentz force. Because of the increased opposition, the flow slows down, flattening the velocity profile and lowering momentum transmission over the boundary layer. The momentum barrier layer becomes thicker because the fluid particles close to the needle surface undergo a larger retarding effect, which is relevant for magnetic hyperthermia and targeted drug delivery systems. The fluid gains internal thermal energy because of this energy being directly injected into it. The fluid’s temperature, therefore, increases noticeably. The combined effects of this magnetic dissipation and viscous dissipation, or friction within the fluid, produce a sharper temperature gradient. This gradient is particularly noticeable in the area around the needle surface. More thermal energy is trapped and dispersed throughout the fluid, thickening the thermal boundary layer. The graphical trends confirm that the Lorentz force plays a more significant role in altering the flow and thermal gradients of HNFs compared to conventional third-grade fluids. Fig. 4 depicts the variation of the third-grade factor $\left( \beta \right)$ on liquid flow and thermal gradients. Due to the diminution in thickness of the boundary layer, the velocity declines, and the thermal boundary layer rises. The fluid’s effective viscosity increases under shear as $\beta$ rises. By acting as an increased friction inside the fluid, this shear-thickening tendency increases the resistance to flow. The velocity profile flattens and the flow speed decreases over the boundary layer as a consequence of the stronger opposition to fluid motion from the greater viscous forces. A higher $\beta$ value results in increased flow resistance, which causes more energy to be dissipated by viscous dissipation. Within the fluid, the effort exerted against the increased viscous forces is immediately transformed into heat. The fluid’s temperature rises as a result of this, acting as an internal heat source. As a result, the temperature gradient steepens and the thermal boundary layer thickens, particularly close to the needle surface where shear rates are greatest. The thermal and momentum boundary layer upsurge as the velocity ratio factor $\left( a \right)$ rises, as exposed in Fig. 5. Longer thermal interaction and improved fluid entrainment are the main causes of the expansion of the momentum and thermal boundary layers with an increasing velocity ratio parameter. To pull a larger volume of fluid and transmit momentum over a longer area, a higher needle surface velocity causes the surrounding fluid to experience more kinematic force and shear stress. This thickens the momentum barrier layer. This enlargement of the hydrodynamic zone directly affects the thermal field because it lengthens the residence period of fluid particles close to the heated needle surface. Thus, both boundary layers expand simultaneously as a result of the convective transfer of momentum and energy being amplified by an increase in the velocity ratio parameter. The effect of needle size $\left( \chi \right)$ on the velocity and thermal gradients are exposed in Fig. 6. The increase in needle size leads to a reduction in both the thermal and momentum boundary layers. Physically, as the needle grows thicker, its surface area increases, and when it encounters the nanofluid particles, the resulting higher drag force ultimately reduces the velocity. Furthermore, when needle stiffness increases, the thickness of the velocity boundary layer increases. The impression of viscous dissipation $\left( {Ec} \right)$ on the thermal gradient is portrayed in Fig. 7. The thermal gradient of both liquids is enhanced as the viscosity of the liquids increases. When the Eckert number rises, the energy produced by viscous friction takes centre stage. Within the boundary layer, this internally produced heat functions as a dispersed volumetric heat source. As a result, the fluid temperature increases far more than it would by conduction and convection alone. Because more energy is held in the fluid, the temperature gradient at the surface becomes steeper and the thermal boundary layer thickens overall. Physically, increasing the liquid viscosity creates wall friction. As a result, the liquid thermal rises and internal heat is produced. The effect of internal heat $\left( H \right)$ on liquid motion is depicted in Fig. 8. The thermal gradient of a moving liquid is quite high. When the needle is moved through the liquid, the temperature rises significantly. A greater internal heat source is indicated by an increase in $H.$ The fluid’s internal thermal energy directly rises as a result of this energy being evenly distributed throughout the fluid volume. In addition to convective and conductive heat transmission, this serves as a dispersed heat source. A steeper thermal gradient close to the surface and an overall thickening of the thermal boundary layer are the results of the increased energy input, which raises the fluid temperature significantly across the boundary layer. Fig. 9 shows the relationship between the Prandtl number $\left( {\Pr } \right)$ and the thermal gradient. The thermal conductivity and thickness of the liquid fall as the $\Pr$ rises, and the rate at which heat is transferred from the needle declines. The slower molecular transmission of energy prevents the heat from the needle surface from penetrating far into the fluid. Fig. 10 demonstrates the dominance of *Nr* on the thermal gradient of a liquid and HNF. The analysis reveals that the thermal gradient improves with rising values of the thermal radiation factor *Nr*​. As *Nr* rises, the intensity of radiative thermal energy transmission becomes more significant, leading to a thermal enhancement within the fluid domain. The depth of thermal impact may be efficiently increased via radiative heat transfer, which can transmit energy directly from hotter areas close to the needle surface to cooler areas farther away. Because the fluid absorbs radiation volumetrically, this increased energy intake raises the temperature significantly across the boundary layer. The greater thermal gradient in the dimensionless representation is the result of the combined action of radiative and conductive transport, which produces a more dispersed temperature field with higher overall temperatures in nuclear cooling and solar thermal systems**.** Fig. 11 demonstrates the effect of varying the thermal ratio factor $\left( {\theta_{w} } \right)$ on the thermal gradient of the HNF and liquid. The thermal ratio of the ambient and wall thermals is given by $\theta_{w} .$ Higher values of the wall thermal ratio factor $\theta_{w}$ ​signifies that the surface thermal of the needle is substantially greater than the ambient fluid temperature. As $\theta_{w}$ enlarges, a steeper thermal gradient develops, resulting in a rise in the thermal gradients of both the base liquid and the hybrid nanofluid. The temperature across the boundary layer rises noticeably as a result of the increased thermal energy delivered into the fluid.

The impression of ${\mathrm{Al}}_{{2}} {\mathrm{O}}_{{3}}$ $\left( {\phi_{1} } \right)$ on flow and energy gradients is described in Fig. 12. It is visible that raising the nanosized-materials volume fraction of liquid improves the thermal and flow gradients. Physically, the NFs density decreases due to the higher value of $\phi_{1} ,$ which consequently grows the velocity and thermal. This impact is the most prominent and immediate. When high-thermal-conductivity nanoparticles (${\mathrm{Al}}_{{2}} {\mathrm{O}}_{{3}}$) are added, the fluid’s effective thermal conductivity rises noticeably. Heat is more effectively transferred away from the needle surface due to this improved conductivity. This results in a significant increase in temperature throughout the boundary layer and a steeper thermal gradient close to the surface because thermal energy is dispersed more quickly and efficiently over a greater volume of fluid. When adding nanoparticles, the main result is generally an increase in density and viscosity, which would normally lower velocity and impede flow. However, since viscosity typically decreases with temperature, the fluid’s effective viscosity is reduced as a result of the substantial temperature increase brought on by the increased thermal conductivity. Fig. 13 illustrates the flow and energy trends in various values of ${\mathrm{TiO}}_{{2}}$ $\left( {\phi_{2} } \right)$ nanosized materials. The velocity is found to decline when the value $\phi_{2}$ is boosted, whereas the thermal upsurges. As a result, with higher values of $\phi_{2} ,$ both the momentum and thermal boundary layers thicken. The presence of ${\mathrm{TiO}}_{{2}}$ nanosized materials affect the thermophysical characteristics of the HNF, resulting in a decrease in flow velocity owing to viscous force and an increment in heat transport due to the enhanced thermal conductivity.

### Fuzzy analysis

The fuzzy temperature profiles were computed by modeling the nanoparticle volume fractions of $\phi_{1}$ and $\phi_{2}$ ​ using a Triangular Fuzzy Number (TFN) defined as [0%, 10%, 20%], as illustrated in Fig. 14. The figure comprises four subplots representing the triangular membership functions for fuzzy temperature distributions corresponding to different values of $\eta ,$ such as 1, 3, 5, and 7. In these plots, the vertical axis denotes the membership values of the fuzzy thermal gradient, while the horizontal axis represents the corresponding fuzzy thermal gradient for varying $\eta .$ Although the input fuzzy volume fractions are symmetric TFNs, the resulting fuzzy temperature profiles are asymmetric TFNs. This asymmetry can be attributed to the nonlinear nature of the governing fuzzy differential equations (FDEs). Furthermore, it is observed that the spread (or width) of the fuzzy thermal profiles for HNFs is notably greater than that of conventional NFs, indicating a higher degree of uncertainty or sensitivity in HNF thermal behavior. Consequently, the HNF shows uncertainty. On the other hand, Fig. 14 shows the comparison of NFs ${\mathrm{Al}}_{{2}} {\mathrm{O}}_{{3}} {\mathrm{/SA}}$ $\left( {\phi_{1} } \right),$ ${\mathrm{TiO}}_{{2}} {\mathrm{/SA}}$ $\left( {\phi_{2} } \right),$ and ${\mathrm{Al}}_{{2}} {\mathrm{O}}_{{3}} {\text{ + Cu/SA}}$ HNFs through membership function for several values of $\eta .$ The nanoparticle volume fraction $\phi_{1}$​ is modeled as a TFN at $\phi_{2} = 0$ represented by black dotted lines in the plots, and when $\phi_{2}$ is TFN then $\phi_{1} = 0$ signifies the blue dotted lines. For the HNF configuration, where both $\phi_{1}$ and $\phi_{2}$​ are non-zero, as depicted using red lines. The HNF exhibits superior thermal performance, as the thermal variation in HNF is more pronounced compared to both individual NFs. This enhanced behaviour can be attributed to the synergistic effect of the combined thermal conductivities of ${\mathrm{Al}}_{{2}} {\mathrm{O}}_{{3}}$ and ${\mathrm{TiO}}_{{2}}$, which collectively promote more efficient thermal energy transmission in the hybrid suspension. When comparing the individual NFs, it is observed that the nanofluid containing $\phi_{1}$ ​ exhibits a higher thermal energy transmission rate than the one with $\phi_{2}$ ​, owing to the intrinsically higher thermal conductivity of ${\mathrm{Al}}_{{2}} {\mathrm{O}}_{{3}} {\mathrm{/SA}}$ compared to and ${\mathrm{TiO}}_{{2}} {\mathrm{/SA}}{.}$ (Table 4).

**Table 4:** Skin friction and Nusselt number for $M, \beta , Pr, Nr, {\phi }_{1}\mathrm{and} {\phi }_{2}$.

$M$	$\beta$	$Pr$	$Nr$	${\phi }_{1}$	${\phi }_{2}$	$Cf$	${Nu}_{x}$
0.01	0.5	0.7	0.1	0.02	0.02	2.62600463	1.23182511
0.03	0.5	0.7	0.1	0.02	0.02	2.62736079	1.23262836
0.05	0.5	0.7	0.1	0.02	0.02	2.62908668	1.23339942
0.01	2	0.7	0.1	0.02	0.02	2.63115979	1.23413814
0.01	4	0.7	0.1	0.02	0.02	2.63355984	1.23484435
0.01	6	0.7	0.1	0.02	0.02	2.62600463	1.2355179
0.01	0.5	1	0.1	0.02	0.02	3.37578341	1.16685059
0.01	0.5	1.5	0.1	0.02	0.02	3.38980726	1.16421601
0.01	0.5	2	0.1	0.02	0.02	3.40395207	1.16153069
0.01	0.5	0.7	0.1	0.02	0.02	3.4182186	1.15879429
0.01	0.5	0.7	0.5	0.02	0.02	3.43260759	1.12934064
0.01	0.5	0.7	0.7	0.02	0.02	3.44711982	1.12627244
0.01	0.5	0.7	0.1	0.01	0.02	4.84625652	1.12292345
0.01	0.5	0.7	0.1	0.03	0.02	4.87273129	1.11951898
0.01	0.5	0.7	0.1	0.05	0.02	4.89943795	1.11605867
0.01	0.5	0.7	0.1	0.02	0.01	5.78028135	1.16153069
0.01	0.5	0.7	0.1	0.02	0.03	5.81516802	1.15879429
0.01	0.5	0.7	0.1	0.02	0.05	5.85037519	1.15601392

## Artificial neural networking (ANN)

Artificial Neural Networks (ANNs) have emerged as powerful computational tools capable of approximating complex nonlinear systems with remarkable accuracy; however, their use in scientific modeling warrants critical examination. While ANNs excel at capturing intricate relationships without explicit governing equations, this strength often comes at the cost of interpretability, making them function as “black boxes” rather than transparent analytical models. In fluid dynamics and thermal energy transmission, for instance, ANNs enable rapid solutions for highly nonlinear problems, yet their reliability heavily depends on the quality and diversity of training data, raising concerns about generalizability beyond the trained domain. The ANN model was developed as a feedforward multilayer perceptron (MLP) with an architecture comprising an input layer of seven neurons (for parameters $M,\,\,\beta ,\,\,\Pr ,\,\,Nr,\,\,\phi_{1} ,\,\,\phi_{2}$​), three hidden layers with ten neurons each using sigmoid activation functions, and a linear output layer for predicting skin friction and heat flux coefficient​. This architecture integrates neurons in hidden layers to train datasets effectively, particularly for predicting drag and flux coefficients. Data obtained from numerical simulations were divided into training (80%), testing (10%), and validation (10%) sets, ensuring systematic model development using Bayesian regularization (BRS) and Levenberg–Marquardt (LMS) algorithms. The Levenberg-Marquardt (LMS) algorithm was selected for its rapid convergence on medium-sized datasets, while the BRS algorithm was employed for its superior ability to prevent overfitting and enhance generalization by incorporating regularization into the optimization process. Model performance was rigorously evaluated based on the Mean Squared Error (MSE) and regression coefficient (R-value) on the testing set, with results demonstrating exceptional predictive accuracy, as evidenced by MSE values approximately $10^{ - 5} - 10^{ - 6}$ and R-values approaching unity, confirming the models’ robustness and reliability as surrogates for the physical system. The model’s performance for flow gradients under simple and hybrid fuzzy NF conditions was assessed through statistical validation, as summarized in Table 5, confirming that the ANN models successfully learned the underlying physics of the system without overfitting and can serve as highly accurate and efficient surrogate models.

**Table 5:** Numerical results exploration via LMS and BRS algorithms for the ANN model.

Algorithms	Quantities	Training	Testing	Epochs	Grad	Performance	Mu
LMS	Skin friction	7.34E-05	1.0791E-05	470	3.48E-04	7.34E-05	1.0E-08
BRS		9.23E-02	2.35E-03	1000	9.33E-05	9.23E-05	0.5
LMS	Nusselt number	1.36E-06	1.67E-06	27	0.00014	1.32E-06	1.0E-08
BRS		1.29E-06	1.66E-06	328	9.89E-08	1.32E-06	0.5

Figure 15(a–h) illustrates the (a-b) error histograms, (c-d) performance analysis, (e-f) training process, and (g-h) regression plots for the skin friction with respect to all factors outlined in Table 4 for LMS and BRS, respectively. The dataset is partitioned such that 80% of the data is allocated for training, while the remaining 20% is reserved for testing and validation. For each acquired sample, the ANN model is employed to predict the output values, which are then compared against the actual target values to assess model accuracy. The error histograms, shown in Fig. 15(a–b), categorize the prediction errors into 20 bins, where the x-axis represents the error intervals and the y-axis indicates the number of data samples within each bin. From the training dataset, approximately 100 samples recorded zero error for the LMS and BRS models, respectively. Fig. 15(c–d) depicts the performance validation plots for assessing the ANN model during testing across each training epoch using the LMS and BRS algorithms. These plots demonstrate how the model iteratively adjusts its internal weights to minimize error. At the initial stages of training, the MSE values are relatively high; however, as training progresses, the error consistently decreases, indicating convergence toward the model’s optimal performance. The dotted lines in the plots represent the point of best validation performance, marking the epoch at which the model achieved its most accurate predictions on the validation dataset. Fig. 15(e–f) shows the training state plots for both LMS and BRS configurations, highlighting key performance metrics including the gradient, optimal number of epochs, and the mu factor. The model’s convergence was evaluated using the MSE, calculated from the squared differences between predicted and actual values. The mu factor acted as a controlling factor, significantly influencing the rate of error convergence during the ANN training process. Figs. 15(g–h) presents the regression plots generated by applying the ANN model across all datasets to evaluate its predictive capability under the LMS and BRS training algorithms. These plots show a correlation coefficient of *R=1* for the training phase and values approaching 1 for both testing and validation phases, which strongly indicates a near-perfect agreement between the predicted and actual outputs. Such results confirm the robustness and accuracy of the developed ANN models, demonstrating their successful execution and generalization across different data subsets. Fig. 16(a–h) provides a comprehensive error analysis between the predicted and target values of the heat flux coefficient, employing error histograms, performance plots, training plots, and regression curves. This evaluation is conducted using both LMS and BRS algorithms, with respect to all the influencing factors listed in Table 4. The results offer critical insight into the accuracy and effectiveness of the ANN model in predicting heat flux coefficient under varying conditions and algorithmic configurations. These graphical representations validate the authenticity of the model and illustrate the linear correlation between the predicted outputs and actual target values. The line-fitting plots consistently demonstrate regression values approaching unity across all examined aspects, thereby confirming that ANN has achieved high predictive accuracy and has performed with remarkable reliability throughout the modeling process.

The bar graph in Fig. 17 illustrates the influence of the third-grade fluid factor $\beta$ on the skin friction for both magnetic $M = 0$ and non-magnetic $M \ne 0$ conditions. An observable trend indicates that as the value of β rises, there is a corresponding decrease in the fluid velocity, highlighting the inverse relationship between the non-Newtonian factor and the flow behavior. Fig. 18 presents the variation of the heat flux coefficient with respect to the thermal radiation factor $Nr$ under both the absence and presence of nanosized-material concentrations. The bar graph reveals that the magnitude of HFC increases as $Nr$ rises for both cases: when the nanosized-material volume fractions are zero and when they are nonzero. This indicates that the thermal radiation effect enhances thermal energy transmission regardless of nanosized-material presence, although the degree of enhancement varies with the concentration levels.

## Conclusion

In this study, the flow and thermal energy transmission behavior of a non-Newtonian third-grade HNF $\left( {{\mathrm{Al}}_{{2}} {\mathrm{O}}_{{3}} {\text{ + TiO}}_{{2}} {\mathrm{/SA}}} \right)$ over a slender thin needle under the influence of magnetic, heat source, nonlinear thermal radiation, viscous dissipation, and heat generation have been comprehensively investigated. The bvp4c approach was used to solve the required equation once it was transformed into a non-dimensional system. For the validation, our findings are in good accord with previously published studies. Nano-sized materials of volume fraction are taken as TFN for the fuzzy thermal energy transmission analysis. An excellent agreement was observed between the ANN-predicted values obtained through both BRS and LMS algorithms and the corresponding target values, thereby validating the accuracy and reliability of the developed model. Some noteworthy findings from the creative contemporary study are as follows:

- The HNF’s $\left( {{\mathrm{Al}}_{{2}} {\mathrm{O}}_{{3}} {\text{ - TiO}}_{{2}} {\mathrm{/SA}}} \right)$ improved heat transfer validates the efficiency of hybrid nanoparticle systems for powerful thermal management applications over single-component nanofluids, with thermal energy transmission rates rising by as much as 15% to 20% respectively.
- As the values of *M* increase, the velocity of the third-grade fluid and HNF decrease while the thermal energy rises, demonstrating the dual capability of magnetic fields to control flow dynamics and enhance heat transfer characteristics in biomedical and industrial applications that require precise thermal regulation.
- Thermal radiation parameter *Nr* and the Eckert number $Ec$ substantially enhance temperature distributions. Both phenomena are particularly significant in high-temperature applications and microscale thermal systems.
- The thermal energy transmission increases with the increase of the temperature ratio parameter $\theta_{w} .$
- Modifications to $M,$ $\beta ,$ $\Pr ,$ $Nr,$ $\phi_{1} ,$ and $\phi_{2}$ enhanced the skin friction coefficient. Nusselt number is improving with the increase of $M,$ $\beta ,$ and $\Pr ,$ while declining with $Nr,$ $\phi_{1} ,$ and $\phi_{2} .$
- A stronger needle velocity ratio parameter decreases the fluid velocity and temperature.
- In the analysis of thermal energy transmission, the $\left( {{\mathrm{Al}}_{{2}} {\mathrm{O}}_{{3}} {\text{ + TiO}}_{{2}} {\mathrm{/SA}}} \right)$ HNFs demonstrate significantly enhanced thermal energy transmission capabilities compared to NFs containing only ${\mathrm{Al}}_{{2}} {\mathrm{O}}_{{3}} {\mathrm{/SA}}$ and ${\mathrm{TiO}}_{{2}} {\mathrm{/SA}}$ NFs, as evidenced by the triangular fuzzy membership functions. Moreover, among the individual NFs, the one containing ${\mathrm{Al}}_{{2}} {\mathrm{O}}_{{3}} {\mathrm{/SA}}$ NFs outperforms that with ${\mathrm{TiO}}_{{2}} {\mathrm{/SA}}$, owing to its superior thermal conductivity characteristics.
- The developed ANN models, trained with both LMS and BRS algorithms, achieved exceptional predictive accuracy for skin friction and Nusselt number, with very low MSE $(10^{ - 5} - 10^{ - 6} )$ and regression values $(R)$ approaching 1. This confirms their efficiency as reliable, rapid computational tools for analyzing this complex system.

### Real-world applications

- Parameter ranges correspond to realistic biomedical applications, including magnetic hyperthermia treatments and drug delivery systems.
- The HNF combination $\left( {{\mathrm{Al}}_{{2}} {\mathrm{O}}_{{3}} {\text{ + TiO}}_{{2}} {\mathrm{/SA}}} \right)$ shows potential for targeted cancer therapy and precision thermal ablation.
- Validated models enable rapid design optimization for advanced biomedical devices.
- Framework applicable to thermal management in aerospace and renewable energy systems.

### Limitations

- Thin needle approximation restricts validity to slenderness ratios ε < 0.3, limiting larger diameter device applications.
- Dilute nanoparticle concentration assumption (φ < 0.05) excludes high-concentration regimes with significant particle interactions.
- Steady-state analysis neglects transient effects important in pulsatile flow conditions.
- The uniform magnetic field assumption may not reflect practical electromagnetic coil configurations.
- Linear temperature-property relationships potentially underestimate nonlinear effects at extreme conditions.
- ANN models require extensive experimental validation across the full parameter space.

### Future directions

- Experimental validation studies are essential for hybrid nanofluid thermal performance and fuzzy uncertainty predictions.
- Extension to pulsatile flow conditions for better physiological application representation.
- Integrating physics-informed neural networks (PINNs) for enhancing physical phenomenon capture.
- Investigation of higher nanoparticle concentrations using modified effective medium theories.
- Development of adaptive fuzzy systems with automatic membership function adjustment.
- Incorporation of non-uniform heat source/sink distributions for realistic device configurations.

## Data Availability

The data supporting the findings of this study are available from the corresponding author upon reasonable request.

## List of symbols

$v$ and $u$: Velocity components (m/s)
*T*: Fluid temperature (*K*)
$$T_{w}$$: Surface temperature (*K*)
$$T_{\infty }$$: Ambient temperature (*K*)
*R = r*: Thin needle radius
$$\theta_{w}$$: Temperature difference
$$\zeta {\text{ - cut}}$$: Fuzzy parameter
$$\delta_{hnf}$$: Electrical conductivity
$H$: Heat source parameter
$$\Pr$$: Prandtl number
$\beta$: Third-grade fluid parameter
$\phi_{1}$ and $\phi_{2}$: The volume percentages of ${\mathrm{Al}}_{2} {\mathrm{O}}_{3}$ and ${\mathrm{TiO}}_{2}$ nanomaterials
$Cf$: Local coefficient of skin friction
$\overline{\theta }\left( {\eta ,\,\zeta } \right)$: Dimensionless fuzzy temperature
$v_{f}$: Kinematic viscosity of fluid $\left( {m^{2} /s} \right)$
$\mu_{hnf}$: Dynamic viscosity of hybrid nanofluid $(kgm^{2} /s)$
$\left( {Cp} \right)_{hnf}$: Heat capacity of hybrid nanofluid
$k_{hnf}$: Thermal conductivity of hybrid nanofluid
$\rho_{hnf}$: Density of hybrid nanofluid $\left( {Kg/m^{3} } \right)$
$$\eta$$: Similarity variable
$$\psi$$: Stream function
$u_{e}$: Free stream velocity (m/s)
${\mathrm{Re}}$: The local Reynolds number
$$N_{r}$$: Thermal radiation
$$Ec$$: Eckert number
$s_{1}$ and $s_{2}$: Solid components
$$M$$: Magnetic Parameter
$Nu_{x}$: Local Nusselt number
$$\theta \left( \eta \right)$$: Dimensionless temperature
$$f^{\prime}\left( \eta \right)$$: Dimensionless velocity
